# Mortality differences and inequalities within and between ‘protected characteristics’ groups, in a Scottish Cohort 1991–2009

**DOI:** 10.1186/s12939-015-0274-8

**Published:** 2015-11-25

**Authors:** A. D Millard, G. Raab, J. Lewsey, P. Eaglesham, P. Craig, K. Ralston, G. McCartney

**Affiliations:** NHS Health Scotland, Meridian Court, 5, Cadogan Street, Glasgow, G2 6QE UK; University of Glasgow (Institute of Health and Wellbeing), 1 Lilybank Gardens, Glasgow, G12 8RZ UK; University of Edinburgh, Edinburgh, EH8 9YL UK

## Abstract

**Background:**

Little is known about the interaction between socio-economic status and ‘protected characteristics’ in Scotland. This study aimed to examine whether differences in mortality were moderated by interactions with social class or deprivation. The practical value was to pinpoint population groups for priority action on health inequality reduction and health improvement rather than a sole focus on the most deprived socioeconomic groups.

**Methods:**

We used data from the Scottish Longitudinal Study which captures a 5.3 % sample of Scotland and links the censuses of 1991, 2001 and 2011. Hazard ratios for mortality were estimated for those protected characteristics with sufficient deaths using Cox proportional hazards models and through the calculation of European age-standardised mortality rates. Inequality was measured by calculating the Relative Index of Inequality (RII).

**Results:**

The Asian population had a polarised distribution across deprivation deciles and was more likely to be in social class I and II. Those reporting disablement were more likely to live in deprived areas, as were those raised Roman Catholic, whilst those raised as Church of Scotland or as ‘other Christian’ were less likely to. Those aged 35-54 years were the least likely to live in deprived areas and were most likely to be in social class I and II. Males had higher mortality than females, and disabled people had higher mortality than non-disabled people, across all deprivation deciles and social classes. Asian males and females had generally lower mortality hazards than majority ethnic (‘White’) males and females although the estimates for Asian males and females were imprecise in some social classes and deprivation deciles. Males and females who reported their raised religion as Roman Catholic or reported ‘No religion’ had generally higher mortality than other groups, although the estimates for ‘Other religion’ and ‘Other Christian’ were less precise.Using both the area deprivation and social class distributions for the whole population, relative mortality inequalities were usually greater amongst those who did not report being disabled, Asians and females aged 35-44 years, males by age, and people aged <75 years. The RIIs for the raised religious groups were generally similar or too imprecise to comment on differences.

**Conclusions:**

Mortality in Scotland is higher in the majority population, disabled people, males, those reporting being raised as Roman Catholics or with ‘no religion’ and lower in Asians, females and other religious groups. Relative inequalities in mortality were lower in disabled than nondisabled people, the majority population, females, and greatest in young adults. From the perspective of intersectionality theory, our results clearly demonstrate the importance of representing multiple identities in research on health inequalities.

**Electronic supplementary material:**

The online version of this article (doi:10.1186/s12939-015-0274-8) contains supplementary material, which is available to authorized users.

## Background

The effect on health of the interaction between socio-economic status (SES) and individual personal characteristics is of great interest internationally [1] and within Scotland [2, 3]. Nine such individual characteristics are legally defined as ‘protected characteristics’ in Scotland under the Equality Act (Scotland) 2010. The protected characteristics are age, sex, race, disability, religion, sexual orientation, transgender status, marriage and pregnancy/maternity. We know that stratification by protected characteristic reveals differences in health, and that for a variety of measures of health, health status in the Scottish population has a linear gradient by Socio-economic status (SES) (levels of area and individual deprivation). But the key question for this research was whether there is evidence of a ‘double health disadvantage’ for particular combinations of SES categories and protected characteristics categories. There are two aspects to this: whether particular protected characteristics have a greater risk of mortality for any given level of SES, and whether particular protected characteristics have a different SES distribution to the rest of the population. Therefore, this research focused on the mortality interaction between the protected characteristics and SES.

The theoretical framework for this research is intersectionality theory. This theory describes a need for a framework which focuses on the needs of people who are members of multiple vulnerable or discriminated-against groups – for example Black females (of course the opposite can also be true – White males can be doubly *advantaged*). Intersectionality theory was first described by Kimberlé Crenshaw, a lawyer, who in 1989 used it to challenge ‘how dominant conceptions of discrimination condition us to think about subordination as disadvantage occurring along a single categorical axis’ [4], Crenshaw concluded:

“If any real efforts are to be made to free Black people of the constraints and conditions that characterise racial subordination, then theories and strategies purporting to reflect the Black community’s needs must include an analysis of sexism and patriarchy. Similarly feminism must include an analysis of race if it hopes to express the aspirations of non-white females.”

As Bauer (2014) puts it

“The explicit theorization and greater application of intersectionality within population health research has the potential to improve researchers' collective ability to more specifically document inequalities within intersectional groups, and to study the potential individual and group-level causes of observed inequalities” [5].

Although a number of reports and journal papers have described socioeconomic health inequalities from the 1980s onwards [6–13] there has been only limited examination in the UK of the potential interaction between individual protected characteristics and socio-economic status (SES) in determining health outcomes (e.g. a recent Scottish study which found that measures of socioeconomic position could not consistently account for ethnic differences in cardiovascular disease [14]).

Differences in relationships between health, ethnicity and socio-economic position are illustrated by examples from the two countries where most research attention has been paid to this: the UK and the USA. In the UK, the majority population experience worse health at similar levels of deprivation than many ethnic minorities [15, 16], while the opposite is true in the USA, [17]. There are also different distributions of ethnic minorities by area deprivation between nations: in England and the USA the distribution of the Black and Asian populations is towards greater deprivation [17, 18] (and towards greater poverty amongst Blacks in the USA [19]). In Scotland, the Asian population is skewed towards the least deprived areas and the Black African population is very skewed towards the most deprived areas [20].

Around the world, and despite frequent discrimination and power imbalances in favour of males, females have ubiquitously lower mortality rates than males [21]. However, the differences in mortality between sexes have varied over time and place – peaking in latter half of the 20^th^ Century for most high income countries as male mortality declined to catch up with that of females [17]. In Scotland males have higher mortality rates than females in every age group, and also experience greater inequalities in mortality [13, 22].

Mortality rates increase exponentially with age, with the exception of the slightly higher mortality rate amongst infants which relates to congenital abnormalities, infection and birth complications [23]. In Scotland, relative inequalities in mortality peak between the ages of 25 and 50 years for both males and females largely because of inequalities in deaths owing to alcohol, drugs, suicide and violence (although the absolute number of deaths in this age range is less than at older ages) [13].

Religion is often associated with ethnicity. For example, in the Scottish context Muslim and Hindu religions are associated with Asian and African ethnicities. However, there is evidence of an independent association between religion and health, not mediated by ethnicity [24–26]. Among majority ethnic (‘White’) groups in Scotland, Catholicism is associated with Irish or Polish ancestry, and an increased risk of worse health in Irish Catholic males and females compared to other religions in Scotland has been identified [27–29].

Two sets of factors were proposed by Krieger and Nazroo as causes of differences in health outcomes for those with protected characteristics: the direct effects of the characteristics of individuals in the groups; and the social discrimination against individuals because they belong to particular groups [30, 31]. The mortality outcomes of those with protected characteristics in Scotland, and the extent to which they may vary by the socio-economic circumstances (which may itself be a consequence of discrimination) within those groups is unknown.

This study aims to determine the risk of mortality for those with protected characteristics in Scotland, to report the risk at each intersection between the stratified protected characteristic and socio-economic category, and to examine inequalities in the gradient in mortality by social class and area deprivation within protected characteristic groups. As indicated above, this provides internationally important evidence about the health patterns associated with the interaction between socio-economic inequality and those with protected characteristics. This pinpointing of ‘double disadvantage’ provides useful evidence to prioritise improvement action.

In our text we have needed to use the terms for ethnic categories that are employed in routine data coding systems in Scotland because they underlie the analysis.

## Methods

### Definitions and data

We defined ‘inequality’ as the gradient in mortality risk across ranked socioeconomic categories (occupational social class or area deprivation). We adopted the definitions of protected characteristics used in the 2010 UK Equality Act (sex, race, age, disability, religion, sexual orientation, transgender status, marriage and pregnancy/maternity).

The Scottish Longitudinal Study (SLS) provided our data. The SLS comprises a 5.3 % sample of the Scottish population drawn from the 1991 Census [32]. The sample included all individuals who were aged <65 years and present at the 1991 Census. The sample were followed to their death, exit (emigration) or censoring (loss to follow-up) at the date of data extract (31^st^ December 2009).

Socio-economic status (SES) was coded using the Registrar General’s six category classification of occupational class [33, 34] and area deprivation using Carstairs 1991 deciles [35]. Protected characteristics groups were categorised in order to produce populations large enough for statistical analysis but which were sufficiently homogeneous to be treated as a single group. Ethnicity was coded from the 2001 Scottish census but sufficient deaths for analyses were only available in the White and Asian groups for analyses (requiring exclusion of the Mixed, Black and Other groupings). The White group contained White Scottish, Other British, Irish, and ‘Any other White’. The Asian group included Indian, Pakistani, Bangladeshi and any other Asian background except Chinese. Chinese were excluded because this group is known to have a different health profile compared to other Asian groups in Scotland [36–38].

Thus ethnicity was coded to ‘White’ and ‘Asian (excluding Chinese)’; age was coded in six groups (0–15, 16–24, 25–34,35–44, 45–54 and 55–64 years), sex to ‘male’ and ‘female’, disability to ‘disabled’ and ‘non-disabled’ (disability defined as a long-term illness limiting daily activities or the work a person could do), and religion to ‘No religion’, ‘Roman Catholic’, ‘Church of Scotland’, ‘Other Christian’, and ‘Other religion’, relating to the religion individuals were ‘raised’ in rather than whether or not they currently practice (see Table 1). We were unable to analyse mortality outcomes for the other protected characteristics because of a lack of available data to classify individuals within the dataset.

**Table 1:** Equality group frequencies by sex

Equality group	Social class		Area deprivation (Carstairs 1991)	
	Females	Males	Females	Males
White	66,901	72,110	112,344	109,226
Asian (excl. Chinese)	217	424	779	856
Disabled	4,590	5,957	9,320	9,782
Non-disabled	62,745	66,917	104,372	101,032
No religion	6,043	7,104	13,884	14,035
Roman Catholic	9,993	9,221	16,606	14,165
Church of Scotland	32,445	33,313	48,317	44,793
Other Christian	4,569	4,327	6,613	5,557
Other religion	255	470	715	831
Total females and males	67,335	72,874	113,692	110,814

### Analysis

There were two analysis stages: calculation of all-cause mortality risk using Cox’s proportional hazards to obtain mortality Hazard Ratios (HRs); then calculation of the relative index of inequality (RII) in mortality risk across the SES gradient for each protected characteristic subgroup. All analyses were conducted separately among males and females except for the analysis examining sex itself, which directly compared measures for males and females.

For the mortality HRs we measured statistical interaction by including a term in Cox Proportional Hazards Regression models for the interaction between mortality risk at each level of socioeconomic status and mortality risk for each stratified protected characteristic against a single baseline (within each sex) of the most deprived Carstairs decile or unemployed people for one of the subgroups within each protected characteristic. For example, all five religious groupings were compared, within each sex, against people who were raised with no religion and who were also in the lowest SES group. That interaction gave the HRs for death over the study period 1991–2009 for each intersection between SES and protected characteristics.

For the calculation of RIIs, in accordance with Bauer [5], we used an additive model. The baseline effect for each protected characteristic on mortality inequality was added to the effect in each of the stratified groupings for protected characteristics to give an overall slope (B) and the exponent of the slope (exp B) for each subgroup of each protected characteristic which was the RII for each group.

In practical terms, in the first models for each protected characteristic we included the characteristic and the interaction between it and the relative rank (within each sex) of the mortality risk in each decile. This interaction term generated the RII. We checked that the model was additive by running a second model containing an additional term for the relative rank. This allowed us to see both the baseline slope for each protected characteristic overall and the slope individually for each religious group. The additivity was checked by adding the individual to the baseline slopes to check they agreed with the overall effects for each religious group in the first model.

We also calculated European age-standardised mortality rates (EASRs) for each protected characteristic and SES-protected characteristic stratum (using the 1976 standard population) for those aged >16 years, for males and females combined and stratified by sex. In this analysis the person-years at risk within the study population were allocated to the appropriate age stratum as individuals aged during the follow-up period.

All HRs for ethnicity were calculated in relation to a single baseline for each sex: White males or females (as appropriate) in the most deprived Carstairs decile (decile 10) for the deprivation analyses, and unemployed people for social class analyses. For the analyses comparing hazards for males and females, females who were in deprivation decile 10 or unemployed (as appropriate for the analysis) were the baseline. For the other protected characteristics (except age group) the baseline categories for the analyses in each sex were the most deprived (for Carstairs) and unemployed people (for social class) for people reporting no religion, and no disability, as appropriate for each characteristic. Age group was treated differently by using the least deprived and social class I as baseline categories within each age group for males and females separately. Chronological age was used as the survival time variable and did not require adjustment [39]. In the analysis by age the Cox regression model was modified to include a time dependent covariate because the age group to which people belonged changed over the follow-up period. By using time dependent covariates and left truncation (to ensure people were entered into population at risk at the appropriate time during follow-up) we were able to measure the association of age-group with mortality risk rather than the effect of aging in the 1991 census cohort (a cohort effect).

Social inequality was estimated using the RII. Each study participant was assigned a relative rank by social class (excluding unemployed) and Carstairs decile; separately for males and females. The relative ranks were calculated for individuals both for their place within the whole population and for their place within the protected characteristic subgroup involved (e.g. amongst Roman Catholic males). The relative ranks for each individual were allocated according to the mid-point ranking of the individuals within the relevant subgroup divided by the total number of individuals (which provided a relative rank between 0 and 1). The relative rank was entered into Cox proportional hazards regression models both as a covariate and as an interaction term with each protected characteristic. This gave us the inequalities slope across each protected characteristic subgroup. The RII was given by the statistical interaction between each subgroup and the relative rank for SES. The exponent of the SES slope within each stratum of each protected characteristic (the natural logarithm; log e) describes the HR and can thus can be interpreted as a relative index of inequality (RII). The RII has the same interpretation as relative risk [40] so an RII of one represents complete equality. The RII is thus a descriptive and a dependent variable, expressing the degree of mortality inequality by SES in each protected characteristic subgroup.

Where the ranks were based on the whole population, the RII measured the effect of the range of deprivation found in the whole population on mortality in each subgroup. The RII calculated using the subgroup relative ranks assessed the effect of the range of deprivation within each subgroup on the mortality within that subgroup. For example: RII for social class for Catholic females was calculated by ranking the subgroup of Catholic females by social class, dividing the ranks by the number of Catholic females with social class assigned to get the relative rank, and then running a Cox model with the relative rank as a term. The RII for social class for Catholic females (based on subgroup relative rankings) was derived in the model results as the exponent of the slope for the relative rank. Individuals who were missing data to allow their classification into SES or protected characteristic were excluded from the analyses. The results of the analyses were summarised in forest plots and heat maps.

All analyses were carried out using the Statistical Package for the Social Sciences (SPSS version 19) and the associated ‘complex samples’ software to facilitate delayed entry analysis (left truncation) in Cox regression. Ethical approval for the study was granted by the University of St Andrews and approval for the analyses was granted by the Privacy Advisory Committee of the Information Services Division (ISD) of NHS National Services Scotland.

## Results

### Distribution of protected characteristics

The distribution of the protected characteristic strata by social class and area deprivation are shown in Additional file 1: Tables S1–S16.

The ethnic population distribution by Carstairs deciles for the whole population was evenly spread over the deciles for the White males and females and U-shaped for Asian males and females (Additional file 1: Tables S1 and S2). However, Asian males and females were more likely to be in social class I and II than White males and females and amongst males were less likely to be in social class V (Additional file 1: Tables S3 and S4). For both males and females, the proportion of the population reporting being disabled increased linearly with increasing deprivation, from 5 % in the least deprived decile to 16 % in the most deprived decile. By social class the highest proportion of disabled males was found in social class III manual (IIIM) (37 %) and the highest proportion of disabled females was in social class III non-manual (IIINM) (27 %) (Additional file 1: Tables S5–S8). Amongst religious groups, Roman Catholic males and females were skewed towards greater deprivation in contrast to those raised in the Church of Scotland as, to an even greater extent, were those who were ‘other Christian’ (Additional file 1: Tables S9 and S10). Although not as stark as the social patterning by area deprivation, higher proportions of males and females reported Roman Catholic or ‘no religion’ in social class V with a reverse patterning for those reporting ‘other Christian’ and ‘other religion’ (Additional file 1: Tables S11 and S12). For age, there were more people aged <16 years in the most and least deprived deciles leading to a U-shaped distribution. For those aged 25–34 years and 55–64 years the distribution across deprivation deciles was flat for males and females. However, for those aged 35–54 years there was a greater proportion resident in the least deprived areas (Additional file 1: Tables S13 and S14). Generally, there were higher proportions of those aged 35–54 years in social class I and II whilst the proportion in social class V increased with age (Additional file 1: Tables S15 and S16).

### Hazards for all-cause mortality by population subgroups

#### Sex

**Fig. 1:**
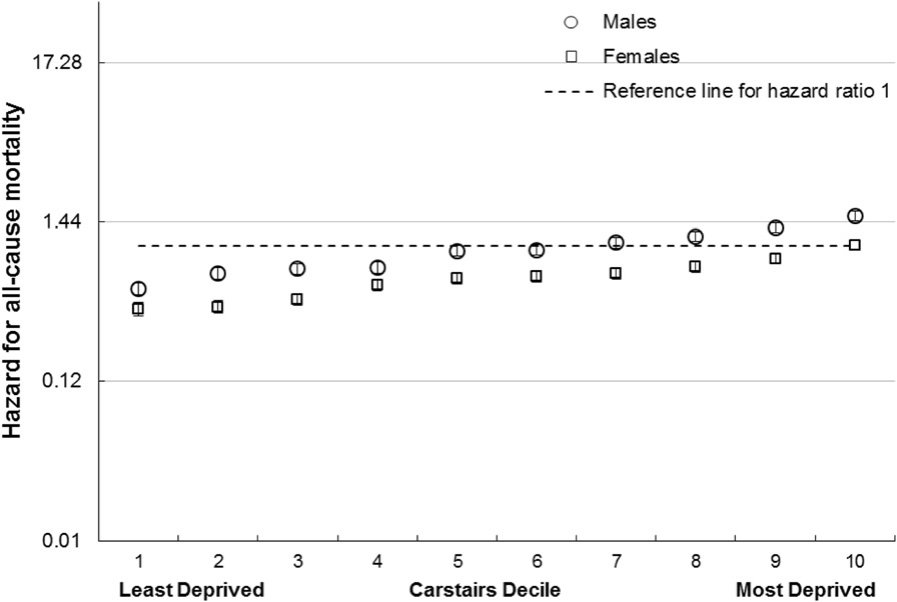
All cause mortality risk by area deprivation and sex. Hazard Ratios and 95 % confidence intervals for all-cause mortality in males and females by area deprivation (compared to females in Carstairs decile 10)

**Fig. 2:**
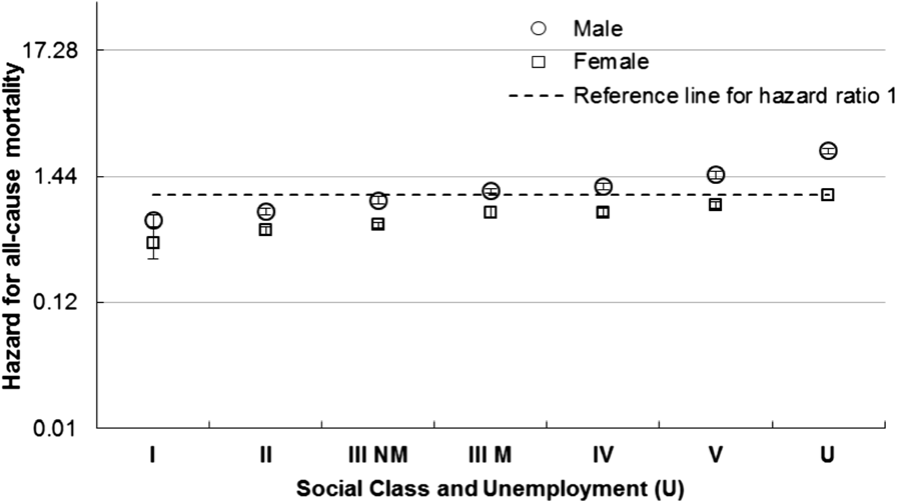
All cause mortality risk by social class, unemployment and sex. Hazard Ratios and 95 % confidence intervals for mortality in males and females by social class and unemployment (compared to unemployed females)

HRs for all-cause mortality between 1991 and 2009 by area deprivation and by social class for males and females in the general population are shown in Figs 1 and 2. There was an increase in mortality from the least to the most deprived for both males and females. The HR in the most deprived males (1.6) was three times higher than the least deprived (0.5) and more than twice as high for females (1.0 compared to 0.4).

The HR for males compared with females after adjustment for age was 1.5 (95 % CI 1.5 to 1.6) with little evidence of an interaction with deprivation; however social class had a more profound impact amongst males than females such that the HR increased to 2.4 (95 % CI 2.3 to 2.5) (Additional file 1: Table S17).

### Ethnicity

**Fig. 3:**
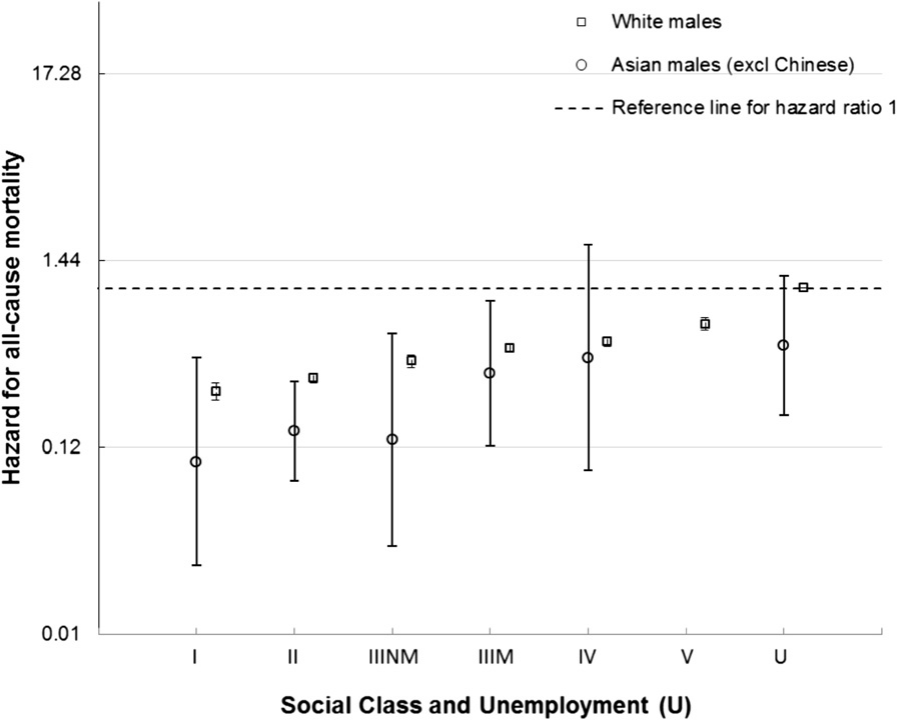
All cause male mortality risk by social class, unemployment and ethnicity. Hazard ratios for mortality by Social class and unemployment in White males and Asian males (compared to White unemployed males)

**Fig. 4:**
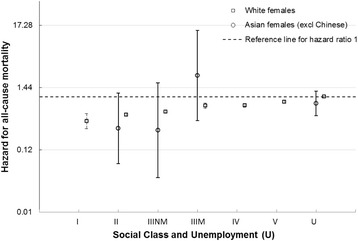
All cause female mortality risk by social class, unemployment and ethnicity. Hazard ratios for mortality by Social class and unemployment in White females and Asian females (compared to White unemployed females)

There was an obvious gradient for Asian males by social class despite the imprecision of the estimates and the lack of deaths amongst Asian males in social class V (which precluded calculation of an HR), with lower HRs in each social class for Asian males compared to White males (Fig. 3). The HR for White females increased across social classes from professionals to unskilled and unemployed (Fig. 4). The HRs for Asian females were imprecise because of the small number of deaths in each group (with too few to calculate HRs for social classes I, IV and V) and as a result no clear patterning was evident.

The HR was higher for White males than Asian males for almost all deprivation deciles, with greater HRs with increasing deprivation for both ethnic groups (Additional file 2: Figure S1). The HRs for Asian males were imprecise because of the small number of deaths. This meant that there was no clear gradient across deprivation deciles for Asian males. Although there was a clear linear gradient for White females across deprivation deciles, with increasing deprivation conferring increased HRs, the patterning for Asian females was less clear (Additional file 2: Figure S2).

The age-adjusted HRs for Asian males and females compared to White males and females were 0.45 (95 % CI 0.31 to 0.66) and 0.78 (95 % CI 0.51 to 1.2) respectively. There was little evidence of an interaction with deprivation and social class, although the estimates were relatively imprecise (Additional file 1: Tables S18 and S19).

### Disability

**Fig. 5:**
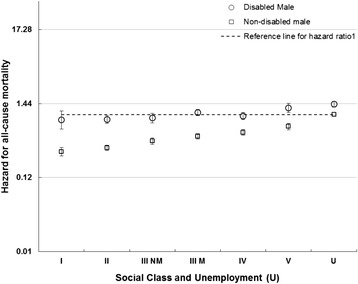
All cause male mortality risk by social class, unemployment and disability. Hazard ratios for mortality in disabled and non-disabled males by social class (compared to unemployed non-disabled males)

**Fig. 6:**
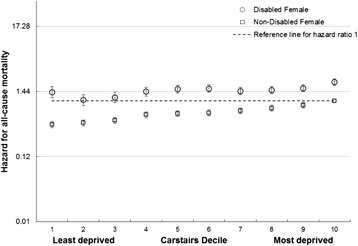
All cause female mortality risk by area deprivation, and disability. Hazard ratios for mortality in disabled and non-disabled females by deprivation (compared to non-disabled females in Carstairs decile 10 – most deprived)

The HRs for disabled and non-disabled females and males were higher with increasing deprivation and lower social class, with higher HRs for those reporting disablement compared to those who did not (Figs 5 and 6 and Additional file 2: Figures S3 and S4).

A similar pattern was seen for disabled and non-disabled males and females across social classes, with increasing hazards from professionals to unskilled and unemployed and higher hazards within each social class for those reporting disablement especially for males (Fig. 5). There was a less clear gradient amongst disabled females (Additional file 2: Figure S4). Amongst males and females, there was an increase in HRs with increasing deprivation for both those reporting disablement and those that did not, although the gradient was less clear for disabled females (Fig. 6 and Additional file 2: Figure S3). For each deprivation category the HR was higher amongst disabled than non-disabled females.

The age-adjusted HRs for disabled females and males were 2.5 (95 % CI 2.5 to 2.6) and 2.4 (95 % CI 2.3 to 2.5) compared to non-disabled females and males respectively. There was evidence of a modest interaction with deprivation and social class for females (reducing the HRs to 2.0 (95 % CI 1.8 to 2.3) and to 2.2 (95 % CI 2.2 to 2.5) respectively) and males (reducing to 1.8 (95 % CI 1.6 to 2.0).and 2.2 (95%CI 2.2 to 2.5) respectively), meaning that the impact of greater deprivation and lower social class were less profound for those reporting disablement than those who did not.

### Religion

The HRs for males and females across deprivation deciles were greater with increasing deprivation for all religions with some exceptions where the estimates were too imprecise to clearly identify a pattern. Those reporting Roman Catholic and ‘no religion’ tended to have the highest HRs within each deprivation decile and social class group (Additional file 2: Figures S5–S8). The age-adjusted HRs across the religious groups were lowest for those reporting being raised as Church of Scotland (HR 0.72, 95 % CI 0.64 to 0.82) and Other Christian (HR 0.53, 95 % CI 0.45 to 0.63). There was a small exacerbation of the differences in mortality risk across religious groups when the interaction with social class was accounted for, but a marked reduction in differences across religious groups when the interaction with deprivation was accounted for (Additional file 1: Tables S22 and S23).

### Age

Additional file 2: Figures S9–S12 describe the HRs by age group, social class and deprivation. The HR for mortality was greater with increasing deprivation for all age groups amongst males, with the gradients generally steeper for younger than older adults (Additional file 2: Figure S9). Amongst females, the HRs for mortality were greater with increasing deprivation for all age groups, with the steepest gradients for those aged 35–54 years and gentler gradients for those <35 years and >75 years (Additional file 2: Figure S10). Across social class groups, the HRs increased from professional to unskilled and unemployed for males, with steeper gradients for younger adults than older adults, and with substantially steeper gradients than those seen across deprivation deciles (Additional file 2: Figure S11). The HRs increased across social class groups for females from professional to unskilled and unemployed for all age groups. The gradients in HRs were even more extreme by age and social class than they were for deprivation, with the steepest gradients for those aged 35–54 years (Additional file 2: Figure S12).

### European age standardised rates (EASRs)

**Fig. 7:**
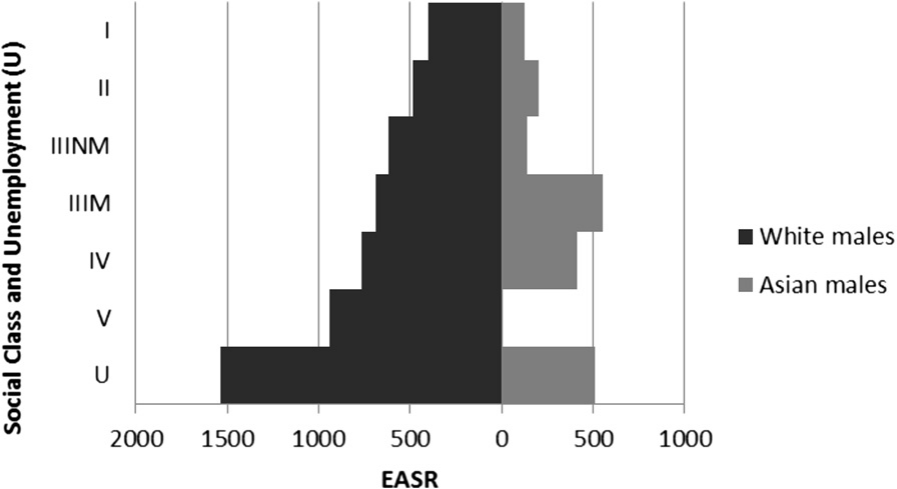
All cause male age standardised mortality rate by social class, unemployment and ethnicity. European Age Standardised Mortality Rates (EASRs) per 100,000 population per year for males within ethnic group by social class and unemployment (1991–2009)

As with the HR data, the EASRs show a general increase with greater deprivation and lower social class overall and for most protected characteristic strata. However, some findings are much more pronounced in this analysis. The EASRs amongst White males and females were markedly higher than those for Asian males and females and the inequality across social categories is much more obvious (Fig. 7 and Additional file 2: Figure S13).

**Fig. 8:**
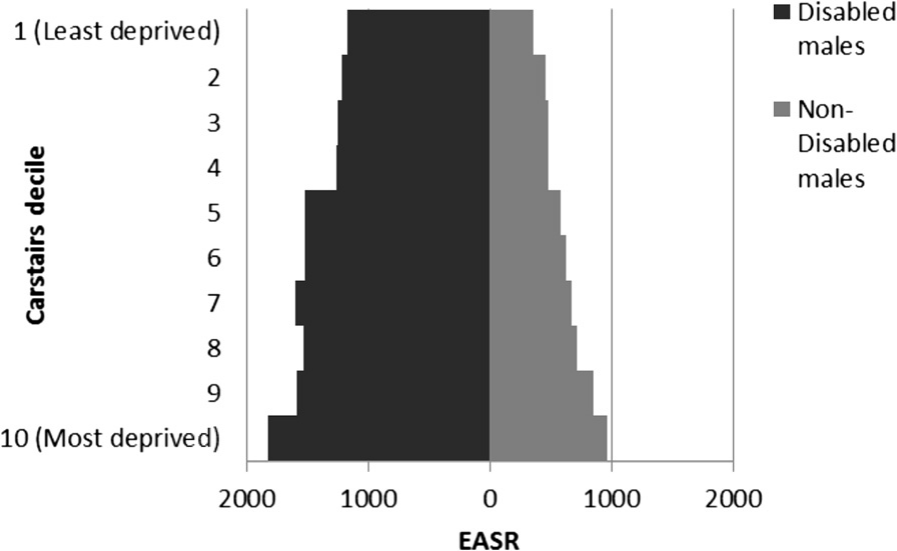
All cause male age standardised mortality rate by area deprivation and disability. EASRs per 100,000 population per year for males by disability and deprivation (1991–2009)

**Fig. 9:**
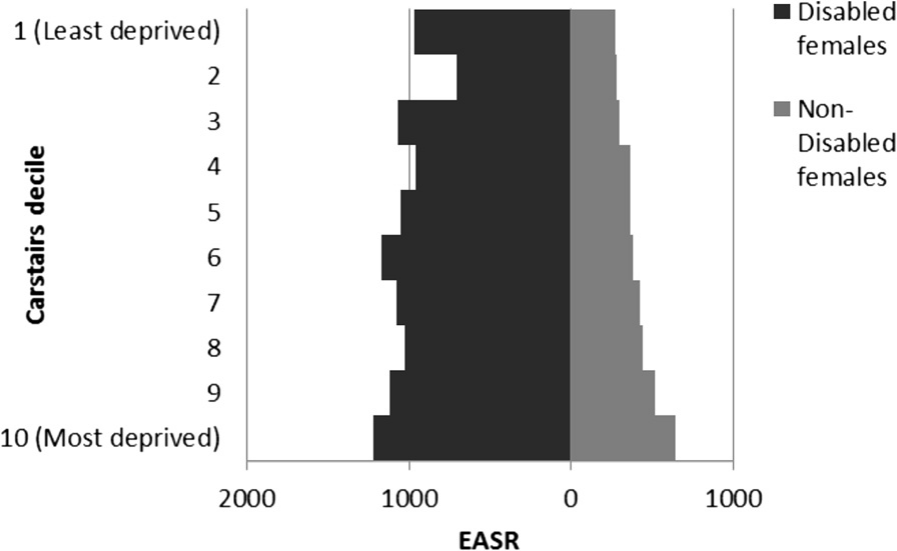
All cause female age standardised mortality rate by area deprivation and disability. European Age Standardised Mortality Rates (EASRs) per 100,000 population for females per year by disability by deprivation (1991–2009)

Similarly, Figs 8 and 9 display the much higher EASRs across deprivation and social class groups for those reporting a disability compared to those who do not (Figs 8 and 9).

The EASRs for religion by social class for males and females shown in Additional file 2: Figures S20 and 21 show higher age standardised mortality rates for Roman Catholic males and females and for males and females of no religion, especially if more socially deprived or unemployed.

### Relative indices of inequality

**Fig. 10:**
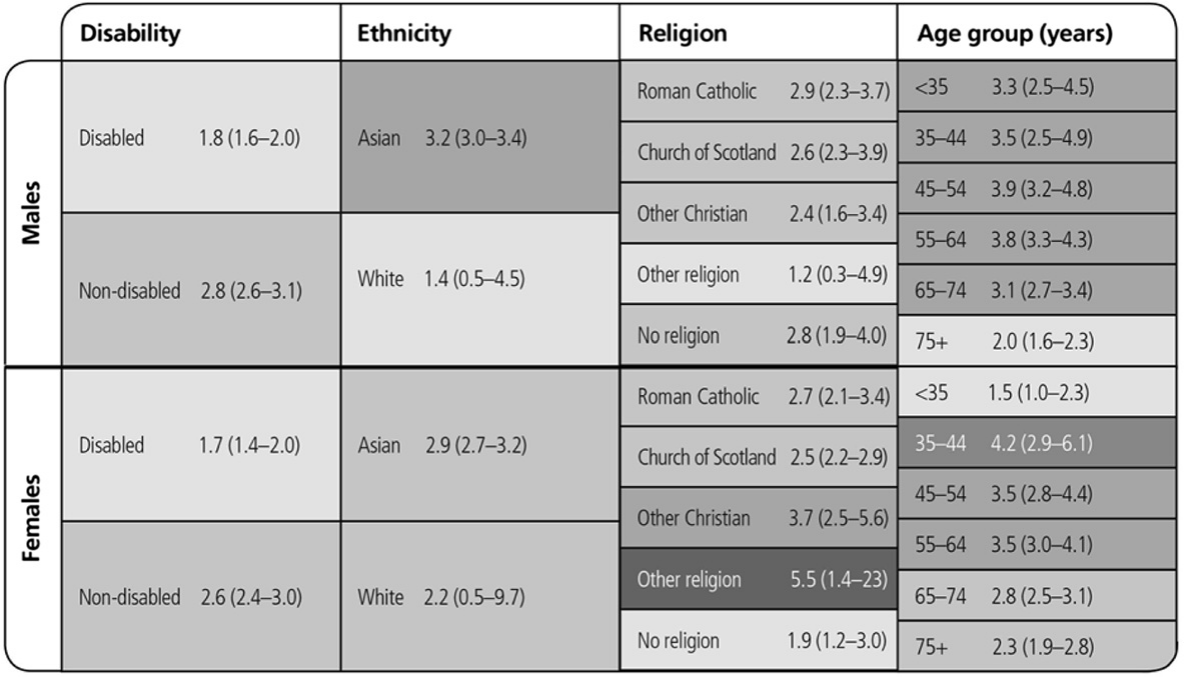
Inequality in risk of death: Heat map by deprivation and protected characteristics groups. Heat map of the RIIs in HRs (95 % CI) by deprivation within the whole population for those aged 16–64 years

There was a deprivation gradient in mortality HRs for all protected characteristic subgroups in Scotland, although some of the RII estimates were very imprecise. The relative inequality (using the whole population distribution of deprivation to allocate the ranks in each subgroup) amongst disabled people was less than the inequality amongst non-disabled people and was greater for Asian than White people, in both males and females. The RIIs for each raised religion group were similar for males (although slightly lower and less precise for those reporting ‘Other religion’). Amongst females, the RIIs were higher for ‘Other Christian’, ‘Other religion’ and lower but imprecise for ‘No religion’. The relative mortality inequalities due to deprivation in the population were consistently high amongst males of all ages, with only a slight decline in inequality in the oldest age group (75+ years). In females, the relative mortality inequalities owing to deprivation in the population were greatest in those aged 35–44 years and were less in those aged <35 years and amongst older adults (Fig. 10).

**Fig. 11:**
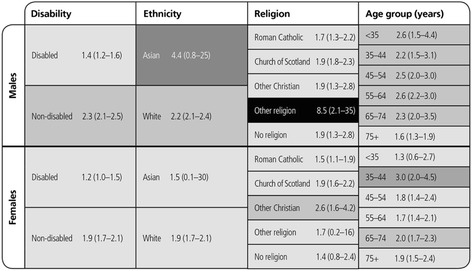
Inequality in risk of death: Heat map by social class and protected characteristics groups. Heat map of RIIs (95 % CIs) of Hazard Ratios by social class within the whole population for those aged 16–64 years in April 1991 (1991–2009)

The RIIs were lower for disabled than for non-disabled males and females based on the social class distributions within the general population. The RIIs for Asian males and females were imprecise and so were difficult to compare with those for White males and females. Within raised religious groups, RIIs were very similar for males with the exception of a higher, but imprecise estimate for those reporting ‘Other religion’ and for females were similar for Roman Catholic, Church of Scotland and ‘No religion’, but were imprecisely higher for ‘Other Christians’ and those reporting ‘Other religion’. Across age groups, males had very similar RIIs except for those aged >75 years where it was lower; but RII’s were higher for females aged 35–44 years than for other groups (Fig. 11).

The RIIs (95 % CIs) for males and for females for area deprivation were 3.2 (95 % CI 3.0 to 3.4) and 2.9 (95 % CI 2.7 to 3.2) and for social class 2.3 (95 % CI 2.1 to 2.4) and 1.9 (95 % CI 1.7 to 2.1) respectively. Our tables showing RII calculated using the hazards ranked within each population subgroup (rather than within the whole population as shown above) are available as Additional file 2: Figures S24 and S25.

## Discussion

### Main findings

We have presented evidence suggesting possible systematic differences in the way SES and protected characteristic subgroups interact to drive differences in mortality inequalities across protected characteristics.

### Distribution of protected characteristic subgroups

The Asian population had a U-shaped distribution across deprivation deciles (in contrast to the flat distribution for the majority ‘White’ population), were more likely to be in social class I and II and (for Asian males only) were less likely to be in social class V. The prevalence of disability rose with increasing deprivation. Those raised as Roman Catholic were more likely to be living in a deprived area and in social class V, in contrast to those raised in the Church of Scotland or as ‘other Christian’. Those aged 35–54 years were least likely to live in deprived areas and were most likely to be in social class I and II.

### Mortality

Males had higher mortality than females, and disabled people had higher mortality than non-disabled people, across all deprivation deciles and social classes. Asian males and females had generally lower mortality hazards than ‘White’ males and females although the estimates for Asian males and females were imprecise in some social classes and deprivation deciles. Males and females who reported their raised religion as Roman Catholic or reported ‘No religion’ had generally higher mortality than other groups, although the estimates for ‘Other religion’ and ‘Other Christian’ were less precise. The impact of social class on mortality was exacerbated amongst males, young adults and within religious strata, but was lower amongst those who were disabled, whilst the impact of deprivation was more muted amongst the disabled, Asians and across religious strata.

### Inequality

Using the area deprivation distribution for the whole population, relative mortality inequalities were greater amongst Asian people and females aged 35–44 years, and smaller for those reporting disablement, the majority ‘White’ population, males by age, females aged <35 years and all people aged >75 years. For the social class distribution in the whole population, a similar pattern was seen with higher RIIs in non-disabled, Asians, females aged 35–44 years and lower for those aged >75 years. The RIIs for the raised religious groupings were generally similar or too imprecise to comment on differences. The relative inequalities using the distributions of deprivation and social class within the equality subgroups were similar to the inequalities using the distributions of the whole population in respect of the higher RIIs. The RIIs using the distributions within each equality subgroup were higher for males than females, those reporting Roman Catholicism, ‘No religion’ and for females reporting ‘Other Christian’ as their raised religion.

### Strengths and weaknesses

#### Strengths

The sample population upon which these analyses are based is drawn from the 1991 Census thereby reducing sampling bias (the 1991 Scottish Census had an estimated response rate of 98.6 % [41]. The Scottish Longitudinal Study percent linkage of records in the 1991 census and the NHS Central Register was 98 % [42]. The NRS death data is of higher validity than NHS death data, for practical purposes 100 % of deaths in Scotland are certified and recorded [43]. Furthermore, there is very little attrition in the cohort over time as there are efforts to account for entry (births and immigrations) and exit (deaths and emigration) events in the sample are enumerated [42]. Excluding deaths and people who moved to England and Wales, sample attrition was 12.2 % (3,2401/265,321) who could not be found in the 2001 census. These will include most of those who moved out of the UK.

Although it hides disease-specific variation, our analysis of all-cause mortality avoids the difficulties in interpretation of overall inequality levels which may result from looking at specific causes, where small inequalities in one disease area may be associated with large inequalities in another.

The EASRs within the cohort are very similar to the Scottish population [44], which provides reassurance that the dataset is broadly representative. Although there are other sources of longitudinal mortality data on ethnicity, the SLS is the best available source linking data on other equality characteristics and subsequent mortality. The data linkage method made possible the creation of a unique and large dataset which allowed us to access information on ethnicity, religion and disability which would not normally be available to studies based on routine data.

The use of Cox regression allowed adjustment for interactions between variables. Age was the survival time variable, so did not need to be adjusted for in the model. By using time dependent covariates and left truncation we were able to measure the association of age-group with mortality risk rather than the effect of aging in the 1991 census cohort (a cohort effect). The linkage also made possible a relatively long follow-up time over which to observe the mortality outcomes.

The analyses by social class have a distinct value above that provided by Carstairs deprivation analyses in that they provide an individual measure of social status for most employed or self-employed people (except those undertaking unpaid domestic duties) rather than make an assumption of individual social status based on the surrounding population (the ecological fallacy).

The two different methods of calculating RII allowed adjustment of the results for each subgroup to the Scottish setting (where the whole population relative rankings were used) and provided a measure for each subgroup in its own right (where the relative rankings within each protected characteristic subgroup were used). There was in general terms a convergence of the patterns resulting from these two methods, but with some interesting sex differences for religion.

The value of calculating proportional hazards is that interactions between protected characteristics, sex and SES were included in the model. This means the hazards are adjusted for these interactions. The model adjusted for age as well because age was the survival time variable. Calculating age-standardised rates for a longitudinal sample adjusts for age but not for the other interactions. The age-standardised rates produced are for mortality over the whole period 1991–2009.

The similar pattern in age standardised rates for males and females to the corresponding mortality hazards provides validating evidence for the mortality hazards calculated using the cox proportional hazards method.

### Weaknesses

Although our overall sample was large, some subgroups were small and had few deaths over the follow-up period, resulting in imprecise HR estimates, the merging of groups (and the consequent danger of obscuring heterogeneity) or their exclusion from the analyses. Furthermore, we did not have data for several of the protected characteristics which we would have liked to have examined.

Social class data were not available for those who were <16 years or for those who had no job in the last 10 years, meaning that we could not examine the patterns for those experiencing periods of unemployment shorter than 10 years, and making the mortality of this group more extreme. Thus they were excluded from the calculations of RII by social class, although we present some results for this unemployed group separately, illustrating their high mortality rates. The lack of independent social class data for many is also a major weakness of the dataset but is a marker of the extent of labour market involvement of at the time of data collection and has been discussed in detail elsewhere [45]. Many of the characteristics we are studying may be related to each other, so we cannot simply imply causal connections. For example lack of a job for 10 years may be the result of a disability and this may be the underlying cause of the high mortality. However, the purpose of this report is not to carry out a complex causal analysis, but to highlight the different gradients of social disadvantage for protected groups.

Some of the RII estimates were particularly imprecise because of the small number of categories for which data were available and the limited distribution of the population of interest across these categories (e.g. amongst Asian males and females).

### How these findings compare to other studies

Our analysis confirms the well-known gradients of mortality risk across deprivation and social class in the Scottish population for both males and females, but for the first time examines these patterns within a limited number of ethnic, disability and raised religion categories.

As outlined in the background, socio-economic circumstances may be differently distributed for ethnic minority groups, which could influence differences in health outcomes [30, 31]. However, this report stratifies the population by social class and deprivation and shows that, within Scotland, it is the majority ethnic (‘White’) population who have the highest mortality risk irrespective of socio-economic circumstances. This differs from the health outcomes seen in England for ethnic minorities and for Black people in the USA (although there are some parallels to the relatively good health experienced by Latino people in that context), although the broad categorisation used here could obscure worse outcomes for the smallest groups. This report considers all-cause mortality. However, it is known that, for some specific causes such as cardiovascular disease, the risks may be higher for some groups (such as people of Indian, Bangladeshi and Pakistani origin) [14]. This contrast emphasises the need to consider overall mortality risk as well as individual causes.

It is recognised that many within ethnic and religious minorities may be immigrants who have arrived in Scotland to work, and who therefore might be subject to the ‘healthy worker effect’ (whereby the healthiest are the most likely to migrate to work) [46]. This may affect some of the health outcomes seen in this study, although subsequent generations may not enjoy the same health benefits [47, 48]. Our results suggest that area deprivation has a lower impact on health in ethnic minority than in White groups, and this is supported by other recent UK research [15].

### Sex differences

There is a well-documented difference in life expectancy between males and females [22]. Males have shorter lives, but females spend more years in ill health (although also have more healthy years than males) [49]. The Scottish life expectancy for males in 2009–10 was 81.0 years in the least deprived quintile, 70.1 years in the most deprived, whilst for females these were 84.2 years and 76.8 years respectively [49]. Consistent with that, our results show greater mortality hazards for males. Although hazards for both males and females increase with deprivation and from social class I to V, the gradient is steeper for males. Data from National Records Scotland (NRS) show that the male-female life expectancy gap increases with increasing area deprivation, which supports our findings [50] p. [17]. Male-female differences are a consistent thread in all of our analyses, and appear to operate slightly differently in some groups as compared to the overall picture for males and females. Asian males and females were not directly compared, but both had generally lower risk than White people of the same sex.

#### Disability

Routine survey data show a gradient of a higher prevalence of long standing illness, health problem or disability with increasing area deprivation in Scotland [51]. The higher disability levels with increasing deprivation support our findings. The same point applies for those assessing their health as bad or very bad [52]. For both males and females, the more deprived the decile the shorter the life expectancy at birth, the shorter the Healthy Life Expectancy (HLE) at birth and the longer the period expected to be spent not in good health [53].

Our findings add to the picture by comparing the socio-economic gradient in mortality risk for disabled and non-disabled people. The lower socioeconomic mortality risk gradient in the former is a new finding. A selection effect where ill-health affects moving into and out of employment, but not social class mobility has been reported recently [54].

The higher mortality rates for the disabled population are not unexpected given that they are selected on the basis of self-reported ill-health for inclusion in that group. However, disablement can also be a focus for discrimination and so it remains unclear the extent to which the health problems that lead to self-reported disablement explain all of the higher mortality in that group.

#### Religion

Raised religion and ethnicity are associated with each another. For example, among White groups in Scotland, Catholicism may be associated with Irish or Polish ancestry and the Muslim and Hindu religions with Asian ethnicity. A higher risk of worse health in Catholic males and females compared to Other religions has been found in earlier research using data from the West of Scotland Twenty-07 study, where after adjusting for social class, higher mortality risks were found between people with one or more Irish Catholic parents compared to those with no Irish Catholic parent [29]. These differences were found across age cohorts and for a range of measures of general and physical health, psychological distress, impairments and disabilities and physical measures such as height, waist-to-hip ratio, and lung function. Males of patrilineal Irish descent in the west of Scotland (who largely had a Roman Catholic heritage) had higher all-cause mortality than the general population [55], but smoking and drinking did not explain much of this difference [56]. Socio-economic position explained about half of the morbidity excess for middle-aged Catholics in Glasgow [27]. Some qualitative data have suggested that institutional sectarianism restricting employment opportunities for (especially older) Catholics may have been a cause of the higher mortality for this group [57].

Males and females who reported ‘No religion’ had high hazards by Carstairs decile, sometimes higher than Roman Catholics. This suggests that any religious belief may be a protective factor against ill health, perhaps through the social structures it provides [58–64]. However, further work is required to fully understand this higher mortality risk because of the possibility of confounding by other factors.

### Age group

Relative mortality inequalities are known to peak in early adulthood in the context of increasing mortality rates with age (with the exception of infants) [22]. This supports our findings, which show the same pattern. According to linked 2001 Census data, older people were at greater risk of being left behind in more deprived areas of decreasing population, as out-migrants tended to be younger [65, 66]. The highest percentages of net out-migration were for age 25–34 years, females and White people. Out-migrants tended to be people of higher social class than in-migrants or stable residents. In-migrants were more likely to be unemployed than out migrants or stable residents.

## Conclusions

Mortality in Scotland is higher in the majority ethnic population (‘White’), disabled people, males, those reporting being raised as Roman Catholics or with ‘no religion’ than in Asians, females and other religious groups. Relative inequalities in mortality were generally lower in disabled than non-disabled people, the majority ethnic population ‘White’, females, and greatest in middle age. Thus, we found some differences in the pattern of inequality in population subgroups, in comparison to the general population.

Furthermore, the importance of representing multiple identities in research on health inequalities is reaffirmed. Mortality and inequalities are not the same for all members of the same religion, ethnic group, and age group. They vary by sex and by SES in ways not always expected, suggesting that protected characteristics can have a moderating effect on SES-based inequalities in health. The needs arising from multiple identities should be recognised as needs and discrimination may differently affect the health for people within different intersections. These analyses pinpoint inequality categories for public health action within the protected characteristics of sex and ethnicity.

Our inequality analyses, providing RIIs, present evidence of overall inequality that can be compared between each protected characteristic subgroup.

## Additional files

Additional file 1:
**Tables S1-S23 provide further  population distributions by SES and HR breakdowns.** (DOCX 43 kb) Additional file 2:
**Figures S1-S25 provide further comparisons of HRs, RIIs and EASRs.** (DOCX 879 kb)
