# Supplementary material for: Mortality differences and inequalities within and between ‘protected characteristics’ groups, in a Scottish Cohort 1991–2009
Source: Int J Equity Health. 2015 Nov 25;14:142. doi: 10.1186/s12939-015-0274-8 (PMC4658811; doi:10.1186/s12939-015-0274-8)
Supplement: Additional file 1: — Tables S1-S23 provide further population distributions by SES and HR breakdowns. (DOCX 43 kb) [file 12939_2015_274_MOESM1_ESM.docx]

**Webtable 1 – Distribution of the male population by Carstairs deprivation and ethnicity**

|  | **Carstairs decile (1=least deprived; 10=most deprived)** | | | | | | | | | | **Total** |
| --- | --- | --- | --- | --- | --- | --- | --- | --- | --- | --- | --- |
|  | **1** | **2** | **3** | **4** | **5** | **6** | **7** | **8** | **9** | **10** |  |
| **White** | 12,544 (12%) | 12,275 (11%) | 11,556 (11%) | 10,769 (9.9%) | 10,382 (9.5%) | 10,296 (9.4%) | 10,328 (9.5%) | 10,368 (9.5%) | 10,311 (9.4%) | 10,437 (9.6%) | 109,266  (100%) |
| **Asian** | 110 (13%) | 104  (12%) | 97  (11%) | 75  (8.8%) | 69  (8.1%) | 46  (5.4%) | 60  (7.0%) | 70  (8.2%) | 90  (11%) | 135  (16%) | 856  (100%) |
| **Non White / Asian** | 82  (12%) | 101 (15%) | 79  (11%) | 70  (10%) | 54  (7.8%) | 52  (7.5%) | 55  (7.9%) | 72  (10%) | 60  (8.7%) | 67  (9.7%) | 692  (100%) |
| **Total** | 12,736  (11%) | 12,480  (11%) | 11,732  (11%) | 10,914  (9.8%) | 10,505 (9.5%) | 10,394 (9.4%) | 10,443 (9.4%) | 10,510 (9.5%) | 10,461 (9.4%) | 10,639 (9.6%) | 110,814  (100%) |

Note: there were 392 males for whom Carstairs decile data were missing.

**Webtable 2 – Distribution of the female population by Carstairs deprivation and ethnicity**

|  | **Carstairs decile (1=least deprived; 10=most deprived)** | | | | | | | | | | **Total** |
| --- | --- | --- | --- | --- | --- | --- | --- | --- | --- | --- | --- |
|  | **1** | **2** | **3** | **4** | **5** | **6** | **7** | **8** | **9** | **10** |  |
| **White** | 12,555 (11%) | 12,224 (11%) | 11,492 (10%) | 11,032 (9.8%) | 10,729 (9.6%) | 10,595 (9.4%) | 10714 (9.5%) | 10,657 (9.5%) | 11,098 (9.9%) | 11,248 (10%) | 112,344 (100%) |
| **Asian** | 91  (12%) | 94  (12%) | 98  (13%) | 62  (8.0%) | 64  (8.2%) | 59  (7.6%) | 54  (6.9%) | 66  (8.5%) | 67  (8.6%) | 124  (16%) | 779  (100%) |
| **Non White/Asian** | 81  (14%) | 60  (11%) | 55  (9.7%) | 57  (10%) | 54  (9.5%) | 47  (8.3%) | 45  (7.9%) | 61  (11%) | 59  (10%) | 50  (8.8%) | 569  (100%) |
| **Total** | 12,727  (11%) | 12,378  (11%) | 11,645  (10%) | 11,151  (9.8%) | 10,847  (9.8%) | 10,701  (9.5%) | 10,813  (9.5%) | 10,784  (9.5%) | 11,224  (9.9%) | 11,422  (10%) | 113,692  (100%) |

Note: there were 219 females for whom carstairs decile data were missing.

**Webtable 3 - Distribution of the male population by social class and ethnicity**

|  | **Social Class** | | | | | | **Total** |
| --- | --- | --- | --- | --- | --- | --- | --- |
|  | **I** | **II** | **IIINM** | **IIIM** | **IV** | **V** |  |
| **White** | 4,178  (5.8%) | 17,388  (24%) | 7,934  (11%) | 24,987  (35%) | 13,276  (18%) | 4,347  (6.0%) | 72,110  (100%) |
| **Asian** | 58  (14%) | 195  (46%) | 79  (19%) | 57  (13%) | 30  (7.1%) | 5  (1.2%) | 424  (100%) |
| **Non White/Asian** | 70  (21%) | 91  (27%) | 43  (13%) | 89  (26%) | 34  (10%) | 13  (4%) | 340  (100%) |
| **Total** | 4,306  (5.9%) | 17,646  (24%) | 8,056  (11%) | 25,133  (35%) | 13,340  (18%) | 4,365  (6.0%) | 72,874  (100%) |

Note: data were missing for 38,672 males who were therefore excluded

**Webtable 4 - Distribution of the female population by social class and ethnicity**

|  | **Social Class** | | | | | | **Total** |
| --- | --- | --- | --- | --- | --- | --- | --- |
|  | **I** | **II** | **IIINM** | **IIIM** | **IV** | **V** |  |
| **White** | 1,159  (1.7%) | 17,130  (26%) | 24,883  (37%) | 5,387  (8%) | 11,485  (17%) | 6,857  (10%) | 66,901  (100%) |
| **Asian** | 18  (8.3%) | 85  (39%) | 72  (37%) | 10  (8%) | 23  (17%) | 9  (10%) | 217  (100%) |
| **Non White/Asian** | 14  (21%) | 83  (27%) | 53  (13%) | 22  (26%) | 35  (10%) | 10  (4%) | 217  (100%) |
| **Total** | 1,191  (1.8%) | 17,298  (26%) | 25,008  (37%) | 5,419  (8%) | 11,543  (17%) | 6,876  (10%) | 67,335  (100%) |

Note: data were missing for 46,793 females who were therefore excluded

**Webtable 5 – Distribution of the male population by Carstairs deciles by disability status**

|  | **Carstairs decile (1=least, 10=most deprived)** | | | | | | | | | | **Total** |
| --- | --- | --- | --- | --- | --- | --- | --- | --- | --- | --- | --- |
|  | **1** | **2** | **3** | **4** | **5** | **6** | **7** | **8** | **9** | **10** |  |
| **Disabled** | 497 (5.1%) | 582 (5.9%) | 625 (6.4%) | 717 (7.3%) | 906 (9.3%) | 968 (9.9% | 1,143 (12%) | 1,336 (14%) | 1,405 (14%) | 1,603 (16%) | 9,782 (100%) |
| **Not disabled** | 12,239 (12%) | 11,898 (12%) | 11,107 (11%) | 10,197 (10%) | 9,599 (9.5%) | 9,426 (9.3%) | 9,300 (9.2% | 9,174 (9.1%) | 9,056 (9.0%) | 9,036 (8.9%) | 101,032 (100%) |
| **Total** | 12,736 (12%) | 12,480 (11%) | 11,732 (11%) | 10,914 (9.8%) | 10,505 (9.5%) | 10,394 (9.4%) | 10,443 (9.4%) | 10,510 (9.5%) | 10,461 (9.4%) | 10,639 (9.6%) | 110,814 (100%) |

Note: data were missing for 392 males who were therefore excluded

**Webtable 6 – Distribution of the female population by Carstairs deciles by disability status**

|  | **Carstairs decile (1=least, 10=most deprived)** | | | | | | | | | | **Total** |
| --- | --- | --- | --- | --- | --- | --- | --- | --- | --- | --- | --- |
|  | **1** | **2** | **3** | **4** | **5** | **6** | **7** | **8** | **9** | **10** |  |
| **Disabled** | 466  (5.0%) | 547 (5.9%) | 607 (6.5%) | 723 (7.8%) | 899 (9.6%) | 967 (10%) | 1,018 (11%) | 1,219 (13%) | 1,367 (15%) | 1,507 (16%) | 9,320 (100%) |
| **Not disabled** | 12,261  (12%) | 11,831 (11%) | 11,038 (11%) | 10,428 (10%) | 9,948 (10%) | 9,734 (9.3%) | 9,795 (9.4%) | 9,565 (9.2%) | 9,857 (9.4%) | 9,915 (9.5%) | 104,372 (100%) |
| **Total** | 12,727  (11%) | 12,378 (11%) | 11,645 (10%) | 11,151 (9.8%) | 10,847 (9.5%) | 10,701 (9.4%) | 10,813 (9.5%) | 10,784 (9.5%) | 11,224 (9.9%) | 11,422 (10%) | 113,692 (100%) |

Note: data were missing for 219 females who were therefore excluded

**Webtable 7 – Distribution of the male population by social class and disability status**

|  | **Social Class** | | | | | |  |
| --- | --- | --- | --- | --- | --- | --- | --- |
|  | **I** | **II** | **IIINM** | **IIIM** | **IV** | **V** | **Total** |
| **Disabled** | 144 (2.4%) | 1,070 (18%) | 563 (9.5%) | 2,227 (37%) | 1397 (24%) | 556  (9%) | 5,957 (100%) |
| **Not disabled** | 4,162 (6.2%) | 16,604 (25%) | 7,493 (11%) | 22,906 (34%) | 11,943 (18%) | 3,809 (5.7%) | 66,917 (100%) |
| **Total** | 4,306 (5.9%) | 17,674 (24%) | 8,056 (11%) | 25,133 (35%) | 13,340 (18%) | 4,365 (6.0%) | 72,874 (100%) |

Note: data were missing for 38,332 males who were therefore excluded

**Webtable 8 – Distribution of the female population by social class and disability status**

|  | **Social Class** | | | | | |  |
| --- | --- | --- | --- | --- | --- | --- | --- |
|  | I | **II** | **IIINM** | **IIIM** | **IV** | **V** | **Total** |
| **Disabled** | 22  (0.5%) | 934 (20%) | 1,250 (27%) | 447 (9.7%) | 1,034 (23%) | 903 (20%) | 4,590 (100%) |
| **Not disabled** | 1,169 (1.9%) | 16,364 (26%) | 23,758 (38%) | 4,972 (7.9%) | 10,509 (17%) | 5,973 (9.5%) | 62,745 (100%) |
| **Total** | 1,191 (1.8%) | 17,298 (26%) | 25,008 (37%) | 5,419 (8.0%) | 11,543 (17%) | 6,876 (10%) | 67,335 (100%) |

Note: data were missing for 46,576 females were therefore excluded

**Webtable 9 - Distribution of the male population by Carstairs deprivation and religion**

|  | **Carstairs decile (1=least, 10=most deprived)** | | | | | | | | | | **Total** |
| --- | --- | --- | --- | --- | --- | --- | --- | --- | --- | --- | --- |
|  | **1** | **2** | **3** | **4** | **5** | **6** | **7** | **8** | **9** | **10** |  |
| **Roman Catholic** | 1,088 (7.7%) | 1,123 (7.9%) | 1,220 (8.6%) | 1,154 (8.1%) | 1,151 (8.1%) | 1,254 (8.9%) | 1,411 (10%) | 1,718 (12%) | 1,840 (13%) | 2,206 (16%) | 14,165 (100%) |
| **Church of Scotland** | 6,026 (14%) | 5,862 (13%) | 5,376 (12%) | 4,878 (11%) | 4,440 (9.9%) | 4,323 (9.7%) | 4,096 (9.1%) | 3,725 (8.3%) | 3,365 (7.5%) | 2,702 (6.0%) | 44,793 (100%) |
| **Other Christian** | 1,029 (19%) | 907  (16%) | 745  (13%) | 666  (12%) | 538 (9.7%) | 454 (8.2%) | 429 (7.7%) | 301 (5.4%) | 290 (5.2%) | 198 (3.6%) | 5,557 (100%) |
| **Other religion** | 137  (17%) | 110  (13%) | 101  (12%) | 73  (8.8%) | 74  (8.9%) | 46  (5.5%) | 54  (6.5%) | 60  (7.2%) | 86  (10%) | 90  (11%) | 831  (100%) |
| **No religion** | 1,364 (9.7%) | 1,423 (10%) | 1,333 (9.5%) | 1,414 (10%) | 1,435 (10%) | 1,488 (11%) | 1,438 (10%) | 1,457 (10%) | 1,415 (10%) | 1,268 (9%) | 14,035 (100%) |
| **Total** | 9,644 (12%) | 9,425 (12%) | 8,775 (11%) | 8,185 (10%) | 7,638 (9.6%) | 7,565 (9.5%) | 7,428 (9.4%) | 7,261 (9.1%) | 6,996 (8.8%) | 6,464 (8.1%) | 79,381 (100%) |

Note: data were missing for 31,825 males who were therefore excluded

**Webtable 10 - Distribution of the female population by Carstairs deprivation and religion**

|  | **Carstairs decile (1=least, 10=most deprived)** | | | | | | | | | | **Total** |
| --- | --- | --- | --- | --- | --- | --- | --- | --- | --- | --- | --- |
|  | **1** | **2** | **3** | **4** | **5** | **6** | **7** | **8** | **9** | **10** |  |
| **Roman Catholic** | 1,248 (7.5%) | 1,298 (7.8%) | 1,329 (8.0%) | 1,267 (7.6%) | 1,362 (8.2%) | 1,544 (9.3%) | 1,633 (9.8%) | 1,933 (12%) | 2,290 (14%) | 2,702 (16%) | 16,606 (100%) |
| **Church of Scotland** | 6,247 (13%) | 6,030 (13%) | 5,570 (12%) | 5,288 (11%) | 4,980 (10%) | 4,597 (9.5%) | 4,477 (9.3%) | 4,129 (8.5%) | 3,756 (7.8%) | 3,243 (6.7%) | 48,317 (100%) |
| **Other Christian** | 1,096 (17%) | 1,049 (16%) | 881  (13%) | 770  (12%) | 698  (11%) | 522 (7.9%) | 520 (7.9%) | 424 (6.4%) | 377 (5.7%) | 276 (4.2%) | 6,613 (100%) |
| **Other religion** | 115  (16%) | 95  (13%) | 80  (11%) | 59  (8.3%) | 67  (9.4%) | 45  (6.3%) | 55  (7.7%) | 52  (7.3%) | 53  (7.4%) | 94  (13%) | 715  (100%) |
| **No religion** | 1,133 (8.2%) | 1,205 (8.7%) | 1,226 (8.8%) | 1,295 (9.3%) | 1,320 (9.5%) | 1,451 (11%) | 1,520 (11%) | 1,549 (11%) | 1,660 (12%) | 1,525 (11%) | 13,884 (100%) |
| **Total** | 9,839 (11%) | 9,677 (11%) | 9,086 (11%) | 8,679 (10%) | 8,427 (9.8%) | 8,159 (9.5%) | 8,205 (9.5%) | 8,087 (9.4%) | 8,136 (9.4%) | 7,840 (9.1%) | 86,135 (100%) |

Note: data were missing for 27,776 females who were therefore excluded

**Webtable 11 – Distribution of the male population by social class and religion**

|  | **Social Class** | | | | | | **Total** |
| --- | --- | --- | --- | --- | --- | --- | --- |
|  | **I** | **II** | **IIINM** | **IIIM** | **IV** | **V** |  |
| **Roman Catholic** | 428  (4.6%) | 1,981  (22%) | 1,025  (11%) | 3,362  (37%) | 1,762  (19%) | 663  (7%) | 9,221  (100%) |
| **Church of Scotland** | 1,911  (5.7%) | 8,739  (26%) | 3,798  (11%) | 11,641  (35%) | 5,653  (17%) | 1,571  (4.7%) | 33,313  (100%) |
| **Other Christian** | 573  (13%) | 1,570  (36%) | 473  (11%) | 1,013  (23%) | 558  (13%) | 140  (3.2%) | 4,327  (100%) |
| **Other religion** | 52  (11%) | 202  (43%) | 84  (18%) | 81  (17%) | 40  (9%) | 11  (2.3%) | 470  (100%) |
| **No religion** | 304  (4.3%) | 1,260  (18%) | 775  (11%) | 2,717  (38%) | 1,538  (22%) | 510  (7.2%) | 7,104  (100%) |
| **Total** | 3,268  (6.0%) | 13,752  (25%) | 6,155  (11%) | 18,814  (35%) | 9,551  (18%) | 2,895  (5.3%) | 54,435  (100%) |

Note: data were missing for 56,771 males who were therefore excluded

**Webtable 12 – Distribution of the female population by social class and religion**

|  | **Social Class** | | | | | | **Total** |
| --- | --- | --- | --- | --- | --- | --- | --- |
|  | **I** | **II** | **IIINM** | **IIIM** | **IV** | **V** |  |
| **Roman Catholic** | 138  (1.4%) | 2,557  (26%) | 3,489  (35%) | 788  (7.9%) | 1,899  (19%) | 1,122  (11%) | 9,993  (100%) |
| **Church of Scotland** | 496  (1.5%) | 8,501  (26%) | 12,654  (39%) | 2,489  (7.7%) | 5,109  (16%) | 3,196  (9.9%) | 32,445  (100%) |
| **Other Christian** | 183  (4.0%) | 1,676  (37%) | 1,615  (35%) | 287  (6%) | 519  (11%) | 289  (6.3%) | 4,569  (100%) |
| **Other religion** | 17  (6.7%) | 84  (33%) | 102  (40%) | 21  (8.2%) | 24  (9.4%) | 7  (2.7%) | 255  (100%) |
| **No religion** | 79  (1.3%) | 1,026  (17%) | 2,332  (39%) | 660  (11%) | 1,384  (23%) | 562  (9.3%) | 6,043  (100%) |
| **Total** | 913  (1.7%) | 13,844  (26%) | 20,192  (38%) | 4,245  (8%) | 8,935  (17%) | 5,176  (9.7%) | 53,305  (100%) |

Note: data were missing for 60,606 females who were therefore excluded

**Webtable 13 – Distribution of the male population by area deprivation and age**

| **Age group in 1991** | **Decile (1=least, 10=most deprived)** | | | | | | | | | | **Total** |
| --- | --- | --- | --- | --- | --- | --- | --- | --- | --- | --- | --- |
|  | **1** | **2** | **3** | **4** | **5** | **6** | **7** | **8** | **9** | **10** |  |
| **>16 years** | 3,320 (12%) | 3,103 (11%) | 2,839 (10%) | 2,540 (9%) | 2,356 (8.5%) | 2,350 (8.4%) | 2,398 (8.6%) | 2,587 (9.3%) | 2,910 (10%) | 3,469 (12%) | 27,872  (100%) |
| **16-24 years** | 1,556 (11%) | 1,518 (10%) | 1,436 (9.7%) | 1,405 (9.4%) | 1,392 (9.4%) | 1,462 (9.8%) | 1,486 (10%) | 1,519 (10%) | 1,536 (10%) | 1,568 (11%) | 14,878 (100%) |
| **25-34 years** | 2,052 (9.5%) | 2,278 (11%) | 2,435 (11%) | 2,260 (11%) | 2,242 (10%) | 2,123 (9.9%) | 2,096 (9.8%) | 2,100 (9.8%) | 2,032 (9.5%) | 1,879 (8.7%) | 21,497 (100%) |
| **35-44 years** | 2,507 (14%) | 2,379 (13%) | 2,115 (12%) | 1,924 (11%) | 1,718 (9.5%) | 1,624 (9.0%) | 1,592 (8.8%) | 1,485 (8.2%) | 1,342 (7.4%) | 1,369 (7.6%) | 18,055 (100%) |
| **45-54 years** | 1,943 (13%) | 1,866 (12%) | 1,667 (11%) | 1,507 (10%) | 1,447 (10%) | 1,418 (9%) | 1,449 (10%) | 1,376 (9.0%) | 1,347 (8.8%) | 1,203 (7.9%) | 15,223 (100%) |
| **55-64 years** | 1,358 (10%) | 1,337 (10%) | 1,243 (9.3%) | 1,279 (9.6%) | 1,350 (10%) | 1,421 (11%) | 1,425 (11%) | 1,443 (11%) | 1,294 (10%) | 1,154 (9%) | 13,304 (100%) |
| **Total** | 12,736 (12%) | 12,481 (11%) | 11,735 (11%) | 10,915 (9.8%) | 10,505 (9.5%) | 10,398 (9.4%) | 10,446 (9.4%) | 10,510 (9.5%) | 10,461 (9.4%) | 10,642 (9.6%) | 110,829 (100%) |

Note: data were missing for 377 males who were therefore excluded

**Webtable 14 – Distribution of the female population by area deprivation and age**

| **Age group in 1991** | **Decile (1=least, 10=most deprived)** | | | | | | | | | | **Total** |
| --- | --- | --- | --- | --- | --- | --- | --- | --- | --- | --- | --- |
|  | **1** | **2** | **3** | **4** | **5** | **6** | **7** | **8** | **9** | **10** |  |
| **>16 years** | 3,149 (12%) | 3,027 (11%) | 2,696 (10%) | 2,464 (9.2%) | 2,327 (8.7%) | 2,245 (8.4%) | 2,289 (8.6%) | 2,383 (8.9%) | 2,733 (10.3%) | 3,340 (12.5%) | 26,653  (100%) |
| **16-24 years** | 1,448 (9.6%) | 1,432 (9.5%) | 1,421 (9.4%) | 1,438 (9.5%) | 1,429 (9.4%) | 1,520 (10%) | 1,497 9.9%) | 1,590 (11%) | 1,641 (11%) | 1,716 (11%) | 15,132  (100%) |
| **25-34 years** | 2,150 (9.4%) | 2,351 (10%) | 2,434 (11%) | 2,290 (10%) | 2,170 (9.5%) | 2,183 (9.6%) | 2,249 (9.9%) | 2,219 (9.7%) | 2,299 (10%) | 2,452 (11%) | 22,797  (100%) |
| **35-44 years** | 2,649 (14%) | 2,370 (13%) | 2,077 (11%) | 1,941 (10%) | 1,799 (9.5%) | 1,640 (8.7%) | 1,688 (9.0%) | 1,616 (8.6%) | 1,583 (8.4%) | 1,485 (7.9%) | 18,848  (100%) |
| **45-54 years** | 1,936 (12%) | 1,778 (11%) | 1,620 (10%) | 1,540 (9.9%) | 1,557 (10%) | 1,528 (9.8%) | 1,457 (9.3%) | 1,493 (9.6%) | 1,448 (9.3%) | 1,231 (7.9%) | 15,588  (100%) |
| **55-64 years** | 1,398 (9.5%) | 1,421 (9.7%) | 1,400 (9.5%) | 1,479 (10%) | 1,567 (11%) | 1,590 (11%) | 1,632 (11%) | 1,480 (10%) | 1,518 (10%) | 1,199  (8%) | 14,684  (100%) |
| **Total** | 12,730 (11%) | 12,379 (11%) | 11,648 (10%) | 11,152 (9.8%) | 10,849 (9.5%) | 10,706 (9.4%) | 10,812 (9.5%) | 10,781 (9.5%) | 11,222 (9.9%) | 11,423 (10%) | 113,702 (100%) |

Note: data were missing for 209 females who were therefore excluded

**Webtable 15 - Distribution of the male population by social class and age**

|  | **Social Class** | | | | | | **Total** |
| --- | --- | --- | --- | --- | --- | --- | --- |
|  | **I** | **II** | **IIINM** | **IIIM** | **IV** | **V** |  |
| **16-24 years** | 245  (2.3%) | 1,145  (11%) | 1,857  (17%) | 3,894  (36%) | 2,673  (25%) | 907  (8.5%) | 10,721  (100%) |
| **25-34 years** | 1,338  (6.7%) | 4,614  (23%) | 2,353  (12%) | 6,689  (34%) | 3,717  (19%) | 1,246  (6.2%) | 19,957  (100%) |
| **35-44 years** | 1,271  (7.5%) | 5,118  (30%) | 1,599  (9.5%) | 5,719  (34%) | 2,370  (14%) | 761  (4.5%) | 16,838  (100%) |
| **45-54 years** | 845  (6.1%) | 3,999  (29%) | 1,168  (8.4%) | 4,970  (36%) | 2,292  (16%) | 681  (4.9%) | 13,955  (100%) |
| **55-64 years** | 607  (5.3%) | 2,798  (25%) | 1,079  (9.5%) | 3,859  (34%) | 2,287  (20%) | 768  (6.7%) | 11,398  (100%) |
| **Total** | 4,306  (5.9%) | 17,674  (24%) | 8,056  (11%) | 25,131  (35%) | 13,339  (18%) | 4,363  (6.0%) | 72,869  (100%) |

Note: data were missing for 38,337 males who were therefore excluded

**Webtable 16 - Distribution of the female population by social class and age**

|  | **Social Class** | | | | | | **Total** |
| --- | --- | --- | --- | --- | --- | --- | --- |
|  | **I** | **II** | **IIINM** | **IIIM** | **IV** | **V** |  |
| **16-24 years** | 124  (1.1%) | 1,508  (14%) | 5,472  (49%) | 1,204  (11%) | 2,423  (22%) | 434  (3.9%) | 11,165  (100%) |
| **25-34 years** | 575  (2.9%) | 5,376  (27%) | 7,417  (38%) | 1,585  (8.1%) | 3,460  (18%) | 1,239  (6.3%) | 19,652  (100%) |
| **35-44 years** | 262  (1.7%) | 4,772  (31%) | 5,264  (34%) | 1,037  (6.8%) | 2,396  (16%) | 1,560  (10%) | 15,291  (100%) |
| **45-54 years** | 140  (1.2%) | 3,359  (28%) | 3,911  (33%) | 901  (7.5%) | 1,796  (15%) | 1,876  (16%) | 11,983  (100%) |
| **55-64 years** | 90  (1.0%) | 2,283  (25%) | 2,936  (32%) | 692  (7.5%) | 1,467  (16%) | 1,767  (19%) | 9,235  (100%) |
| **Total** | 1,191  (1.8%) | 17,298  (26%) | 25,000  (37%) | 5,419  (8.0%) | 11,542  (17%) | 6,876  (10%) | 67,326  (100%) |

Note: data were missing for 46,585 females who were therefore excluded

**Webtable 17 – HRs for males compared with females testing for interactions with deprivation and social class**

|  | **HRs without interactions (95% CI)** | | | **HR with deprivation interaction**  **(95% CI)** | **HR with Social class interaction**  **(95% CI)** |
| --- | --- | --- | --- | --- | --- |
|  | **All cases*** | **Excluding those without Carstairs data** | **Excluding those without Social class data** |  |  |
| **Females (Baseline)** | 1.0 | 1.0 | 1.0 | 1.0 | 1.0 |
| **Males** | 1.5  (1.5-1.6) | 1.5  (1.5-1.6) | 1.5  (1.5-1.6) | 1.6  (1.5-1.7) | 2.4  (2.3-2.5) |
| **Number of cases** | 225,117 | 224,506 | 168,600 | 224,506 | 168,600 |

*includes those with missing Carstairs/social class data. Note – all analyses are age-adjusted.

**Webtable 18 – HRs for Asian males compared to White males testing for interactions with deprivation and social class**

|  | **HRs without interactions (95% CI)** | | | **HR with deprivation interaction**  **(95% CI)** | **HR with Social class interaction**  **(95% CI)** |
| --- | --- | --- | --- | --- | --- |
|  | **All cases*** | **Excluding those without Carstairs data** | **Excluding those without Social class data** |  |  |
| **White males** | 1.0 | 1.0 | 1.0 | 1.0 | 1.0 |
| **Asian males** | 0.45  (0.31-0.66) | 0.46  (0.32-0.66) | 0.42  (0.28-0.63) | 0.31  (0.11-0.86) | 0.46  (0.18-1.2) |
| **Number of cases** | 110,508 | 110,122 | 81,414 | 110,122 | 81,414 |

*includes those with missing Carstairs/Social class data. Note – all analyses are age-adjusted.

**Webtable 19 – HRs for Asian females compared to White females testing for interactions with deprivation and social class**

|  | **HRs without interactions (95% CI)** | | | **HR with deprivation interaction**  **(95% CI)** | **HR with Social class interaction**  **(95% CI)** |
| --- | --- | --- | --- | --- | --- |
|  | **All cases*** | **Excluding those without Carstairs data** | **Excluding those without Social class data** |  |  |
| **White females** | 1.0 | 1.0 | 1.0 | 1.0 | 1.0 |
| **Asian females** | 0.78  (0.51-1.2) | 0.78  (0.51-1.2) | 0.83  (0.53-1.3) | 0.59  (0.19-1.8) | 0.76  (0.47-1.2) |
| **Number of cases** | 113,338 | 113,123 | 86,380 | 113,123 | 86,380 |

*includes those with missing Carstairs/Social class data. Note – all analyses are age-adjusted.

**Webtable 20 – HRs for disabled females compared to non-disabled females testing for interactions with deprivation and social class**

|  | **HRs without interactions (95% CI)** | | | **HR with deprivation interaction**  **(95% CI)** | **HR with Social class interaction**  **(95% CI)** |
| --- | --- | --- | --- | --- | --- |
|  | **All cases*** | **Excluding those without Carstairs data** | **Excluding those without Social class data** |  |  |
| **Non-disabled females** | 1.0 | 1.0 | 1.0 | 1.0 | 1.0 |
| **Disabled females** | 2.5  (2.4-2.6) | 2.5  (2.4-2.6) | 2.5  (2.4-2.6) | 2.0  (1.8-2.3) | 2.3  (2.2-2.5) |
| **Number of cases** | 113,911 | 113,692 | 86,735 | 113,692 | 86,735 |

*includes those with missing Carstairs/Social class data. Note – all analyses are age-adjusted.

**Webtable 21 – HRs for disabled males compared to non-disabled females testing for interactions with deprivation and social class**

|  | **HRs without interactions (95% CI)** | | | **HR with deprivation interaction**  **(95% CI)** | **HR with Social class interaction**  **(95% CI)** |
| --- | --- | --- | --- | --- | --- |
|  | **All cases*** | **Excluding those without Carstairs data** | **Excluding those without Social class data** |  |  |
| **Non-disabled males** | 1.0 | 1.0 | 1.0 | 1.0 | 1.0 |
| **Disabled males** | 2.5  (2.4-2.6) | 2.4  (2.3-2.5) | 2.4  (2.3-2.5) | 1.8  (1.6-2.0) | 1.4  (1.3-1.6) |
| **Number of cases** | 111,206 | 110,814 | 81,865 | 110,814 | 81865 |

*includes those with missing Carstairs/Social class data. Note – all analyses are age-adjusted.

**Webtable 22 - HRs for males of different religions compared to men reporting No religion testing for interactions with deprivation and social class**

|  | **HRs without interactions (95% CI)** | | | **HR with deprivation interaction**  **(95% CI)** | **HR with Social class interaction**  **(95% CI)** |
| --- | --- | --- | --- | --- | --- |
|  | **All cases*** | **Excluding those without Carstairs data** | **Excluding those without Social class data** |  |  |
| **No religion** | 1.0 | 1.0 | 1.0 | 1.0 | 1.0 |
| **Roman Catholic** | 0.87  (0.76-1.0) | 0.87  (0.76-1.0) | 0.86  (0.75-0.99) | 0.96  (0.66-1.4) | 0.80  (0.64-0.99) |
| **Church of Scotland** | 0.72  (0.64-0.82) | 0.72  (0.64-0.82) | 0.72  (0.63-0.81) | 1.0  (0.72, 1.5) | 0.64  (0.52-0.78) |
| **Other Christian** | 0.53  (0.45-0.63) | 0.53  (0.45-0.63) | 0.52  (0.44-0.62) | 0.73  (0.39-1.3) | 0.46  (0.35-0.61) |
| **Other religion** | 0.74  (0.47-1.2) | 0.75  (0.48-1.2) | 0.76  (0.48-1.2) | 1.0  (0.24-4.1). | 0.71  (0.37-1.4). |
| **Number of cases** | 86,251 | 86,135 | 66,616 | 86,135 | 66,616 |

*includes those with missing Carstairs/social class data. Note – all analyses are age-adjusted.

**Webtable 23 - HRs for females of different religions compared to men reporting No religion testing for interactions with deprivation and social class**

|  | **HRs without interactions (95% CI)** | | | **HR with deprivation interaction**  **(95% CI)** | **HR with Social class interaction**  **(95% CI)** |
| --- | --- | --- | --- | --- | --- |
|  | **All cases*** | **Excluding those without Carstairs data** | **Excluding those without Social class data** |  |  |
| **No religion** | 1.0 | 1.0 | 1.0 | 1.0 | 1.0 |
| **Roman Catholic** | 1.0  (0.92-1.1) | 1.0  (0.92-1.1) | 1.0  (0.92-1.1) | 0.77  (0.57-1.0) | 0.86  (0.65-1.1) |
| **Church of Scotland** | 0.74  (0.67-0.81) | 0.74  (0.67-0.81) | 0.74  (0.67-0.82) | 0.67  (0.50, 0.89) | 0.93  (0.71-1.2) |
| **Other Christian** | 0.56  (0.49-0.64) | 0.56  (0.49-0.64) | 0.56  (0.49-0.64) | 0.49  (0.27-0.89) | 0.63  (0.40-0.98) |
| **Other religion** | 0.50  (0.33-0.76) | 0.50  (0.33-0.76) | 0.50  (0.33-0.77) | 0.18  (0.28-1.2). | 0.16 (0.02-1.1) |
| **Number of cases** | 79,532 | 79,381 | 59,344 | 79,381 | 59,344 |

*includes those with missing Carstairs/social class data. Note – all analyses are age-adjusted.
