# Supplementary material for: Mortality differences and inequalities within and between ‘protected characteristics’ groups, in a Scottish Cohort 1991–2009
Source: Int J Equity Health. 2015 Nov 25;14:142. doi: 10.1186/s12939-015-0274-8 (PMC4658811; doi:10.1186/s12939-015-0274-8)

**Webfigures**

**Webfigure 1 - Hazard ratios for mortality by deprivation decile in White and Asian males (compared to White males in decile 10, most deprived)***


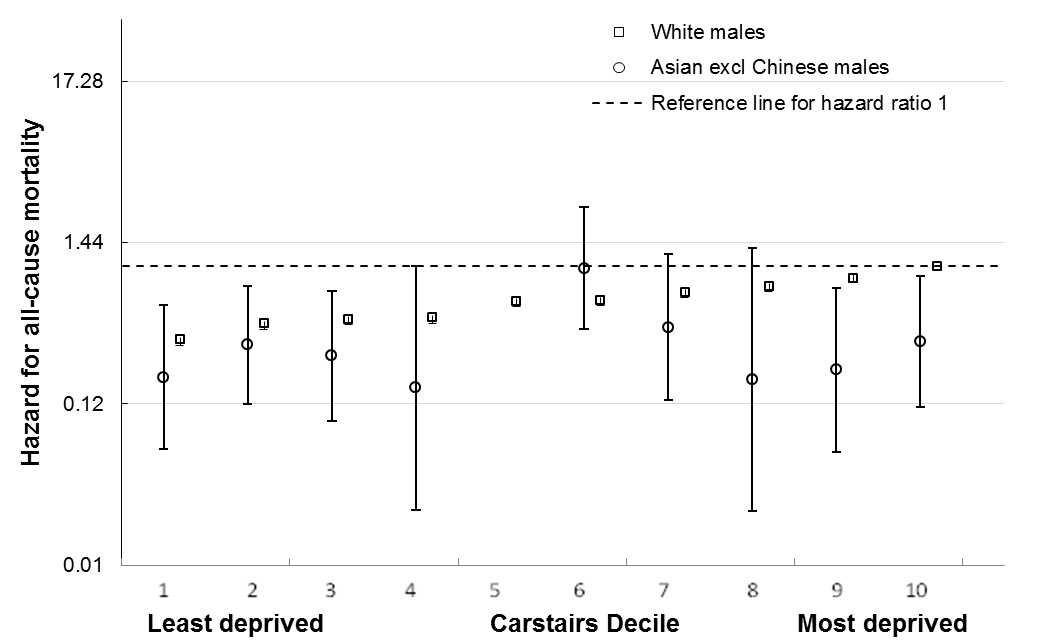


*there were too few deaths in decile 6 for Asians to calculate a HR

**Webfigure 2 - Hazard ratios for mortality by deprivation decile in White females and Asian females (compared to White females in decile 10 – most deprived)**


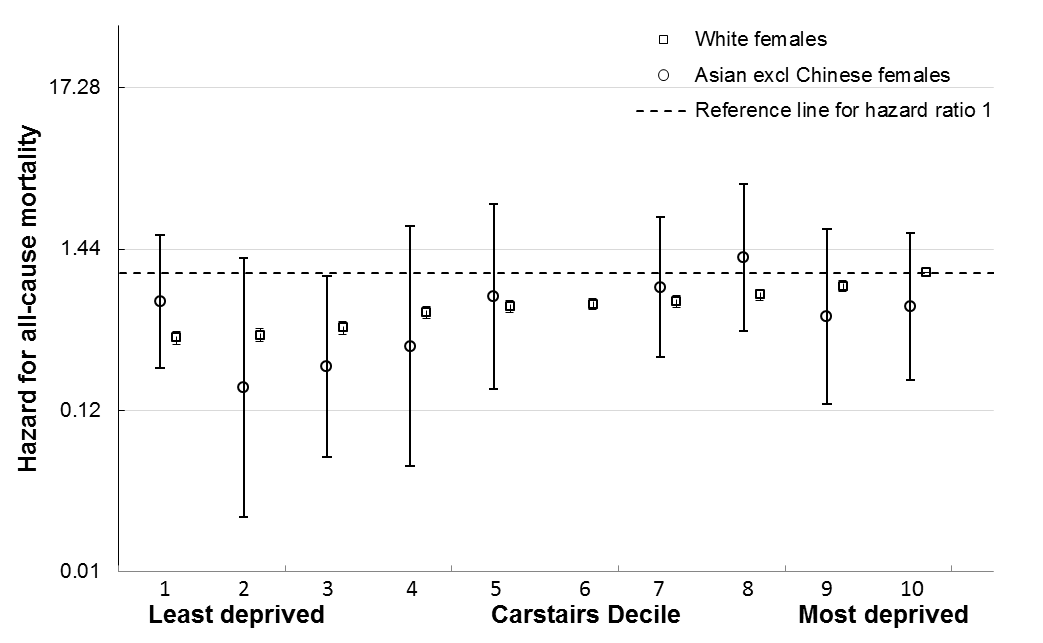


**Webfigure 3 - Hazard ratios for mortality in disabled and non-disabled males by deprivation (compared to non-disabled males in Carstairs decile 10 – most deprived)**


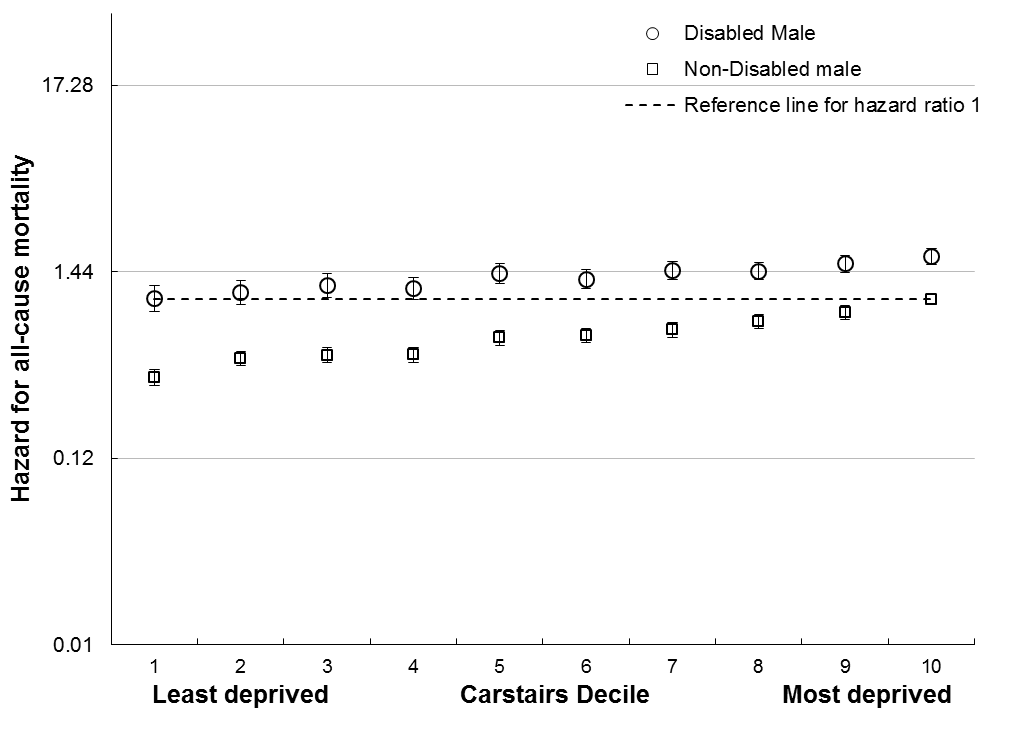


**Webfigure 4 - Hazard ratios for mortality in disabled and non-disabled females by social class (compared to unemployed non-disabled females)**


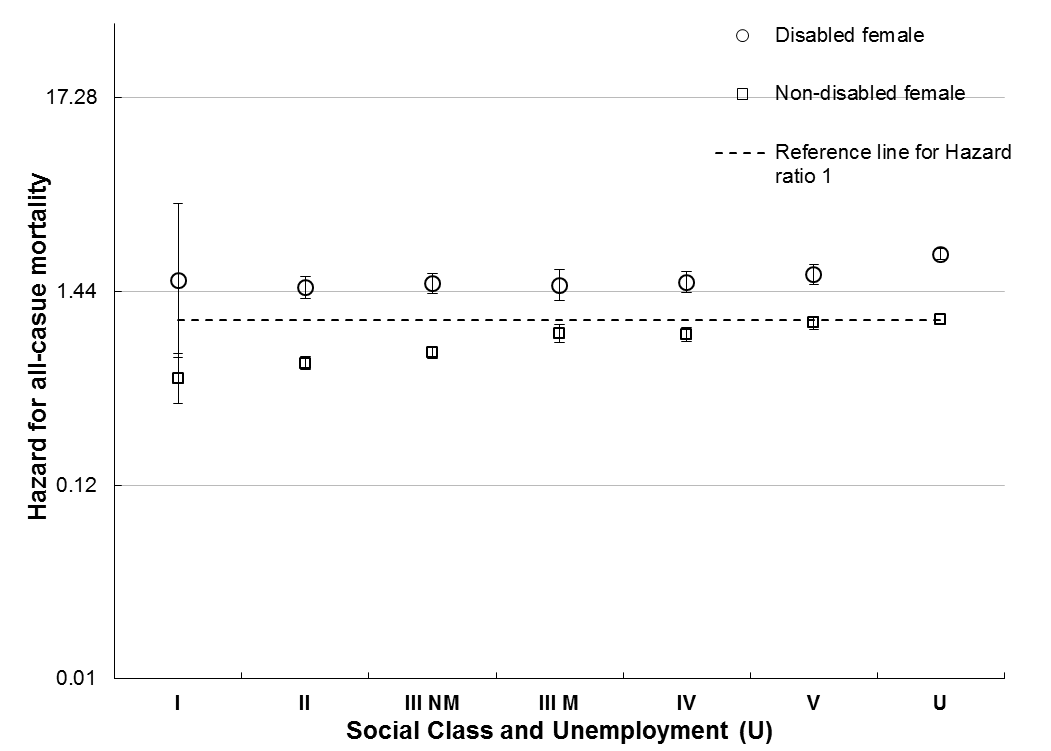


**Webfigure 5 - Hazard Ratios for mortality by religion in males by deprivation (compared to males of No religion in Carstairs decile 10 – most deprived)**


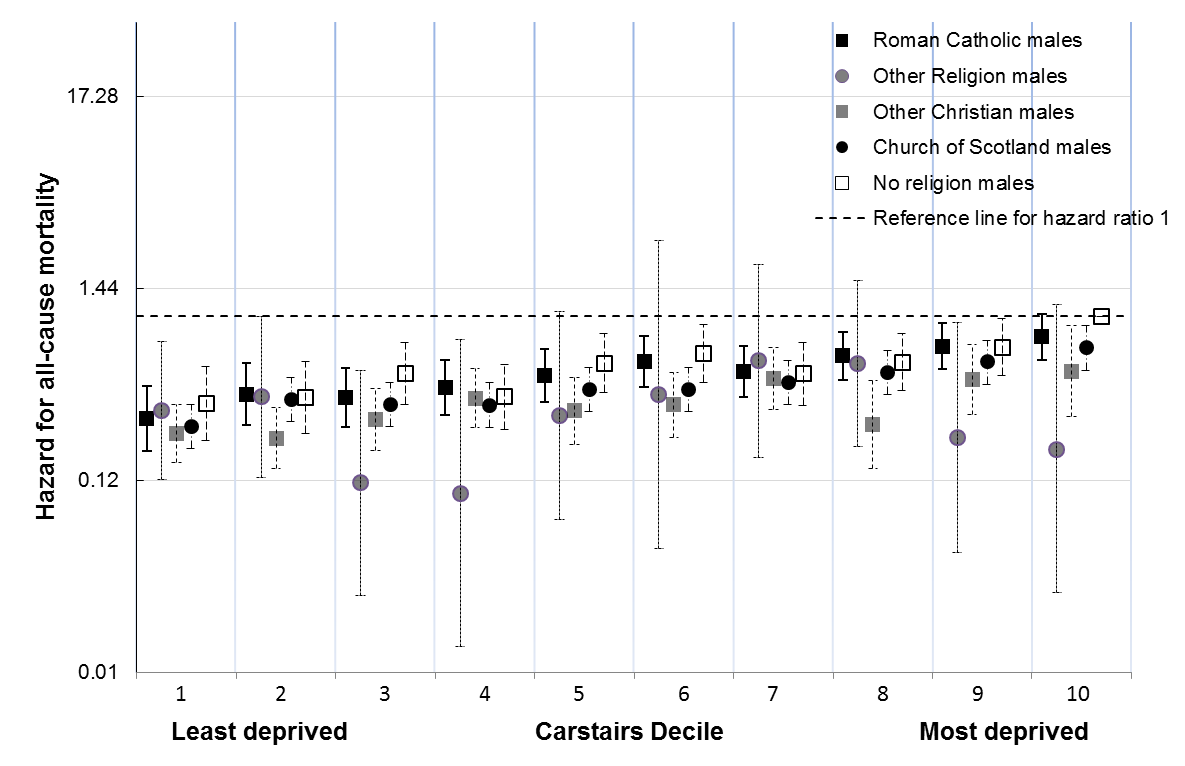


**Webfigure 6 - Hazard Ratios for mortality by religion in females by deprivation (compared to females of No religion in Carstairs decile 10 – most deprived)**

**
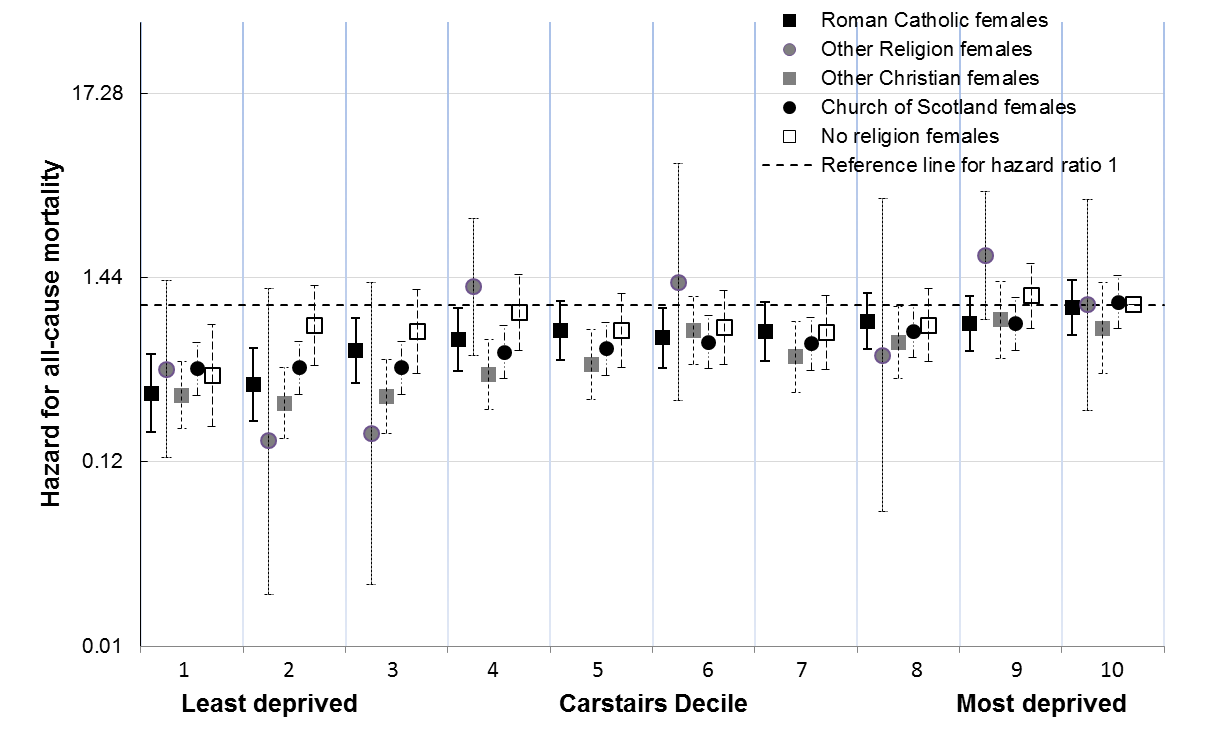
**

**Webfigure 7 - Hazard ratios for mortality in males by religion by social class (compared to unemployed males of No religion)**


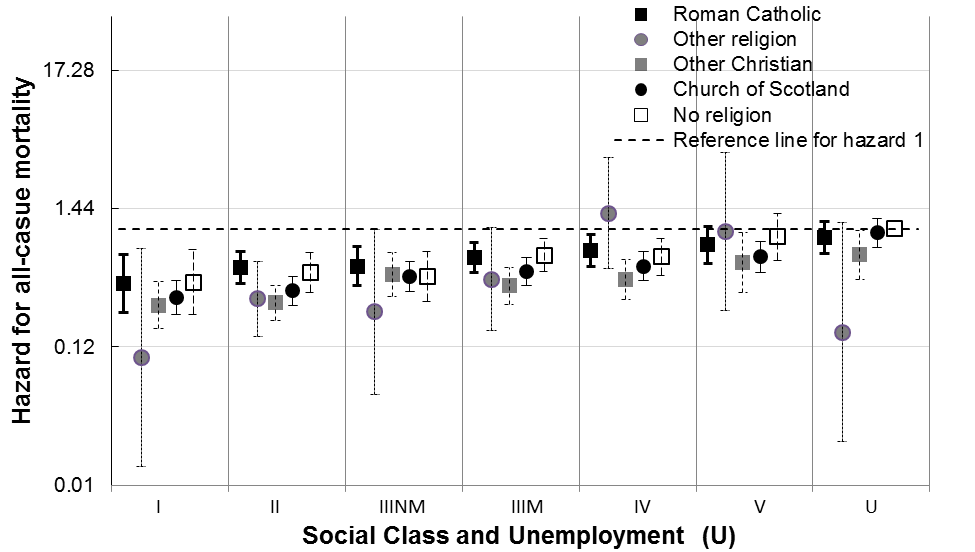


**Webfigure 8 - Hazard ratios for mortality in females by religion by social class (compared to unemployed females of No religion)**


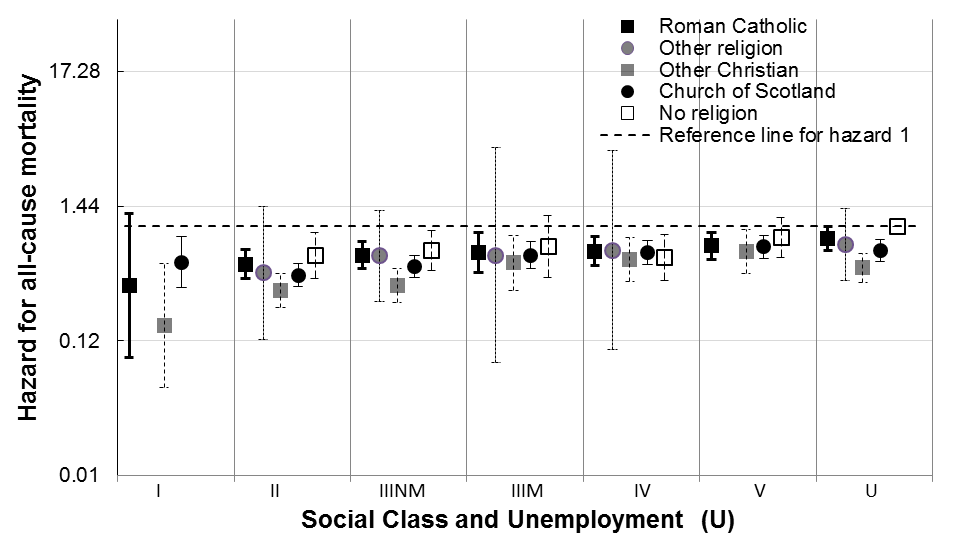


**Webfigure 9 - Hazard ratios for mortality in males by age group by Carstairs 91 decile (compared to Decile 1 (least deprived) males in each age group)**


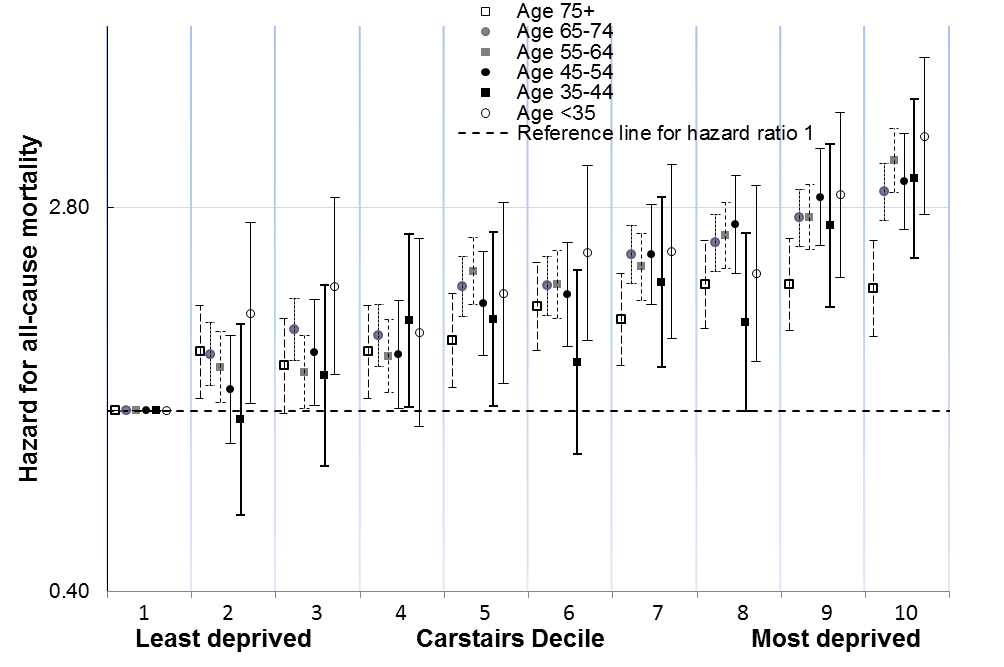


**Webfigure 10 - Hazard ratios for mortality in females by age group by Carstairs 91 decile (compared to decile 1 (least deprived) females in each age group)**


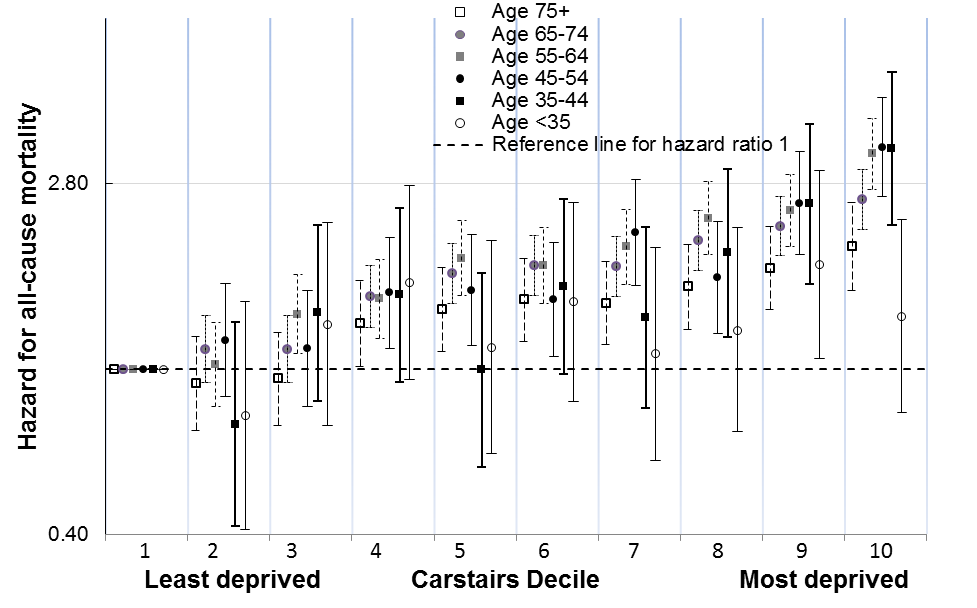


**Webfigure 11 - Hazard Ratios for mortality in malesby age group by social class (compared to social class I males in each age group)**


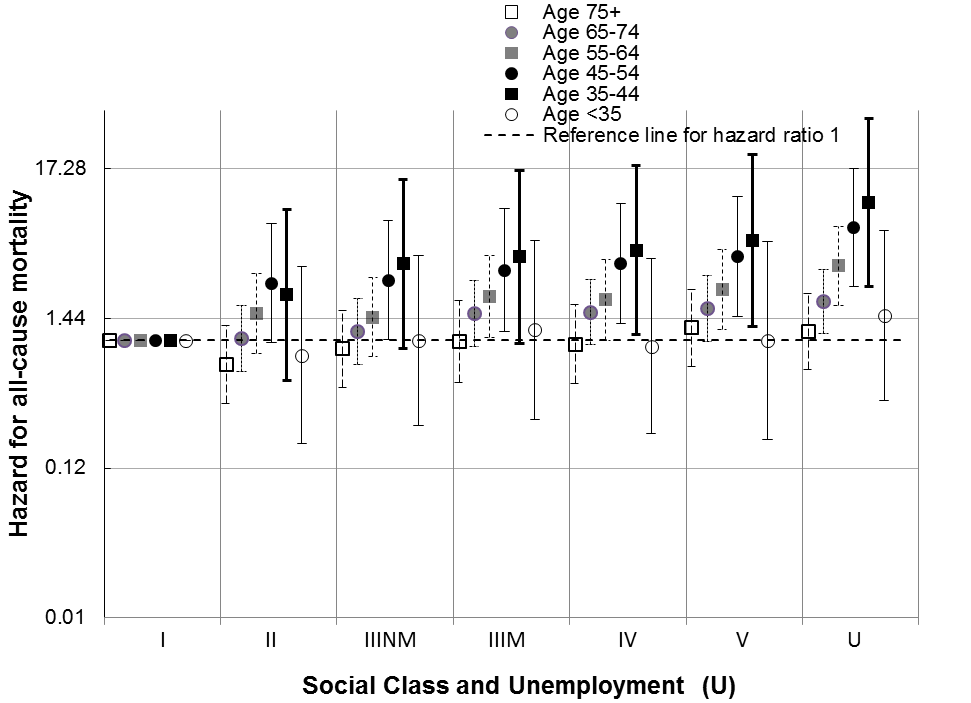


**Webfigure 12 - Hazard Ratios for mortality in females by age group by social class (compared to social class I females in each age group)**


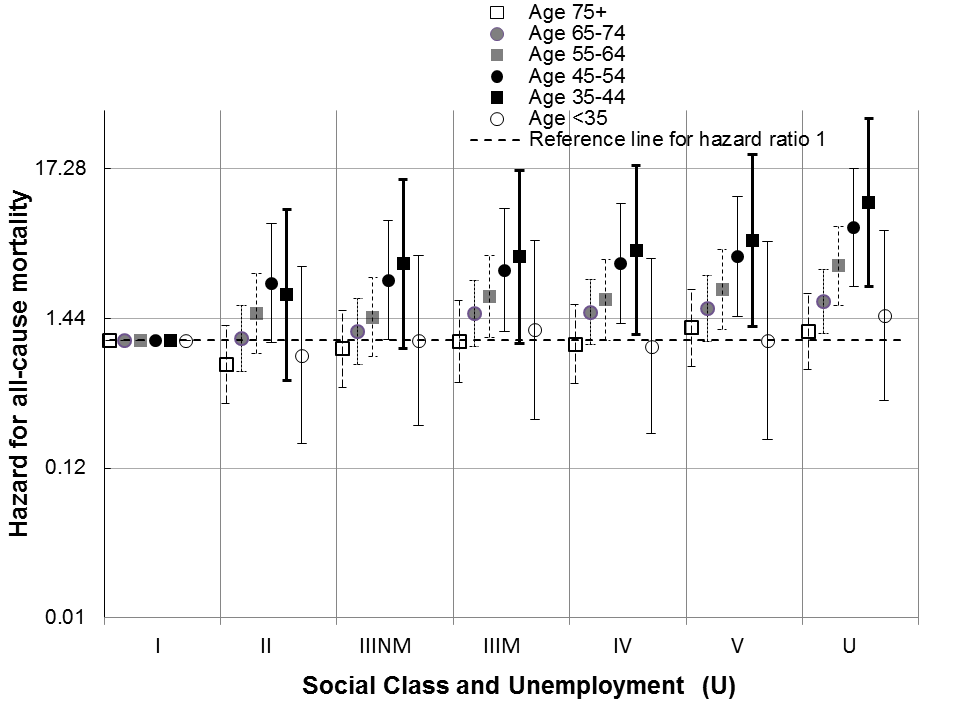


**Webfigure 13: European Age Standardised Mortality Rates (EASRs) per 100,000 population per year for females within ethnic group by social class and unemployment (1991-2009)**


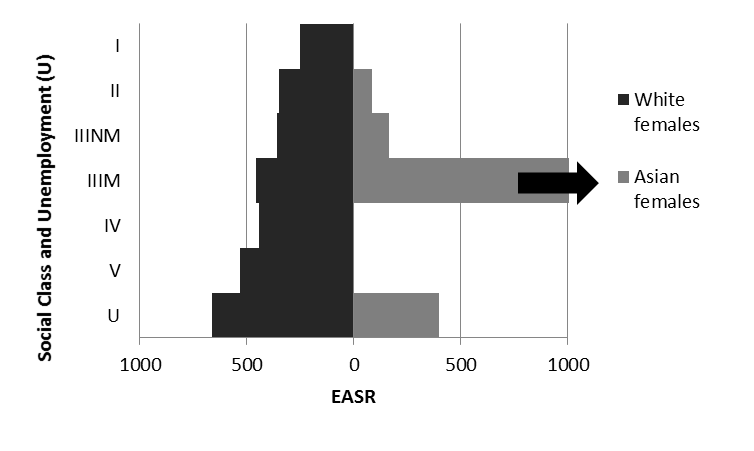


*There were too few deaths in these categories to facilitate the calculation of mortality rates amongst Asian females. Note - the extremely high EASR for Asian females in social class IIIM is a very imprecise estimate as there were only 10 individuals in this stratum and should therefore not be over-interpreted.

**Webfigure 14: European Age Standardised Mortality Rates (EASRs) per 100,000 population for males per year by within ethnic group by deprivation (1991-2009)**

**
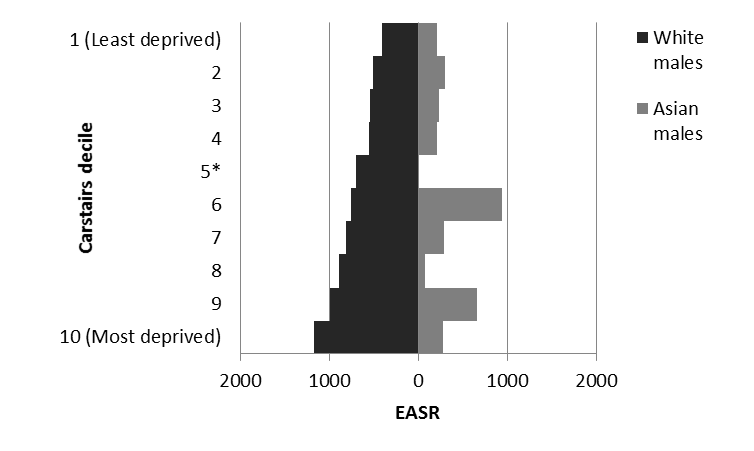
**

*There were too few deaths to facilitate calculation of rates for Asian men in this stratum

**Webfigure 15: European Age Standardised Mortality Rates (EASRs) per 100,000 population for females per year by within ethnic group by deprivation (1991-2009)**

**
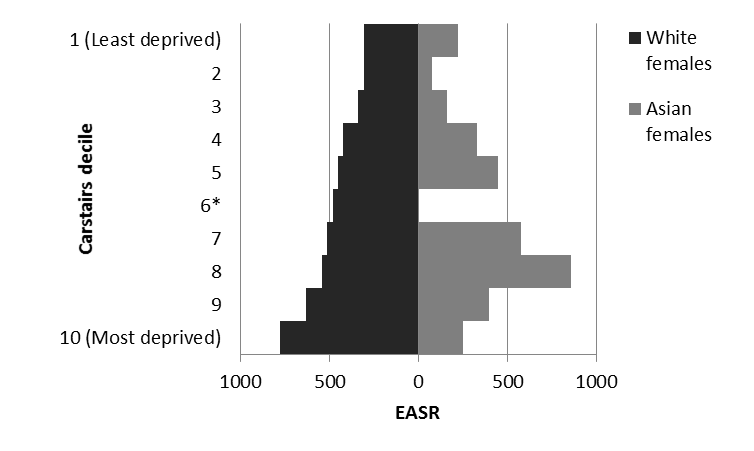
**

*There were too few deaths in this stratum for the calculation of rates for Asian women

**Webfigure 16: European Age Standardised Mortality Rates (EASRs) per 100,000 population per year by social class and unemployment (1991-2009)
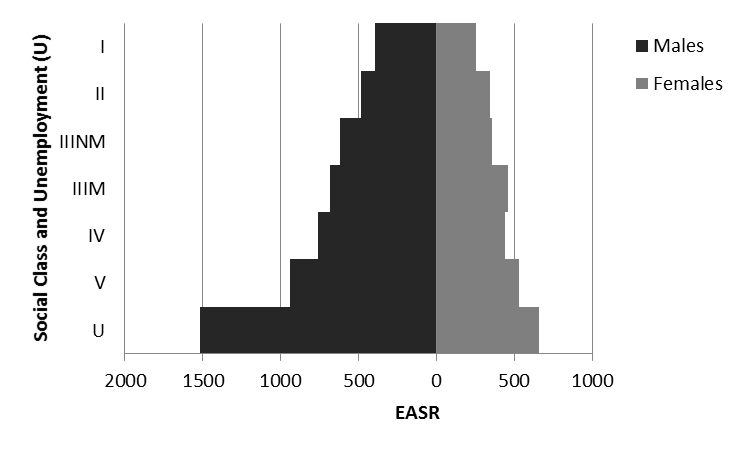
**

**Webfigure 17: European Age Standardised Mortality Rates (EASRs) per 100,000 population per year by deprivation (1991-2009)
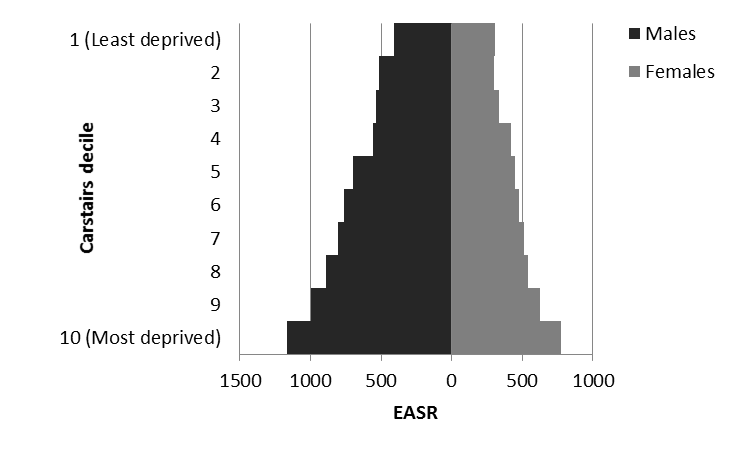
**

**Webfigure 18: European Age Standardised Mortality Rates (EASRs) per 100,000 population per year for males within disability group by social class and unemployment (1991-2009)**

**
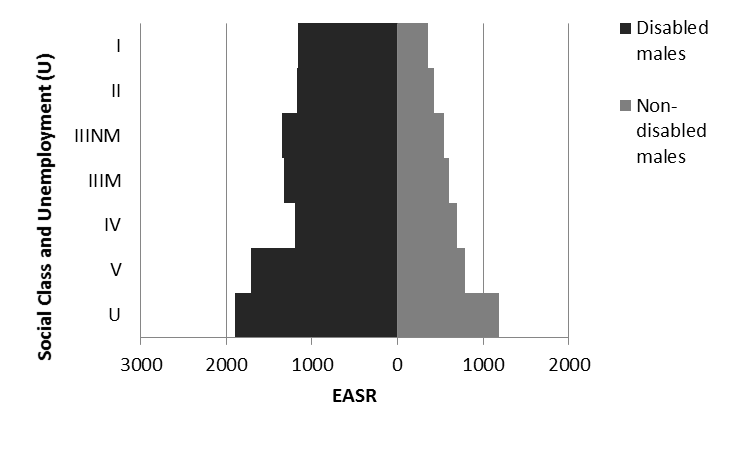
**

**Webfigure 19: European Age Standardised Mortality Rates (EASRs) per 100,000 population per year for females within disability group by social class (1991-2009)**

**
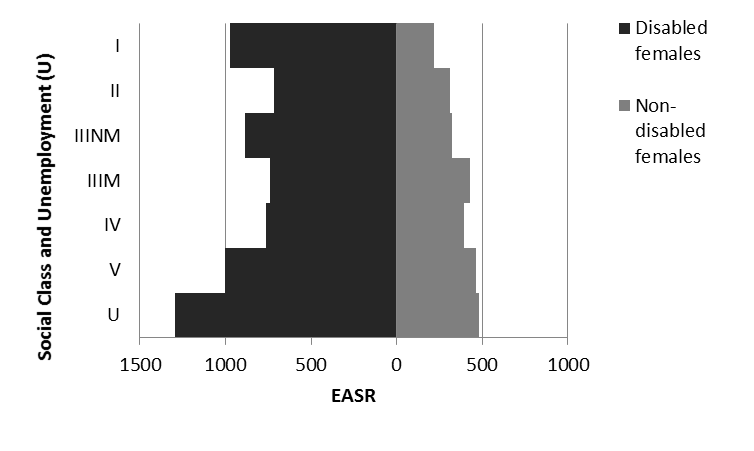
**

**Webfigure 20: European Age Standardised Mortality Rates (EASRs) per 100,000 population per year for males within religious group by social class (1991-2009)**

| **Social Class** | **Roman Catholic** | **Church of Scotland** | **Other Christian** | **Other religion** | **No religion** |
| --- | --- | --- | --- | --- | --- |
| **I (Professional)** | 252 | 298 | 265 | 72 | 275 |
| **II (Managerial/technical)** | 457 | 323 | 233 | 201 | 373 |
| **IIINM (Skilled non-manual)** | 598 | 401 | 448 | 164 | 546 |
| **IIIM (Skilled manual)** | 499 | 431 | 357 | 341 | 524 |
| **IV (Partly skilled)** | 671 | 492 | 370 | 790 | 491 |
| **V (Unskilled)** | 623 | 545 | 493 | 1,272 | 803 |
| **Unemployed** | 832 | 900 | 680 | 112 | 838 |

**Webfigure 21: European Age Standardised Mortality Rates (EASRs) per 100,000 population per year for females within religious group by social class (1991-2009)**

| **Social Class** | **Roman Catholic** | **Church of Scotland** | **Other Christian** | **Other religion** | **No religion** |
| --- | --- | --- | --- | --- | --- |
| **I (Professional)** | 167 | 268 | 66 | * | * |
| **II (Managerial/technical)** | 256 | 225 | 175 | 178 | 230 |
| **IIINM (Skilled non-manual)** | 298 | 257 | 189 | 228 | 276 |
| **IIIM (Skilled manual)** | 284 | 324 | 337 | 178 | 296 |
| **IV (Partly skilled)** | 299 | 323 | 258 | 202 | 282 |
| **V (Unskilled)** | 428 | 353 | 372 | * | 615 |
| **Unemployed** | 436 | 367 | 271 | 287 | 552 |

*There were too few deaths in this group to calculate age-standardised mortality rates

**Webfigure 22: European Age Standardised Mortality Rates (EASRs) per 100,000 population per year for males within religious group by deprivation (1991-2009)**

| **Deprivation decile** | **Roman Catholic** | **Church of Scotland** | **Other Christian** | **Other religion** | **No religion** |
| --- | --- | --- | --- | --- | --- |
| **1 (Least deprived)** | 307 | 275 | 255 | 269 | 407 |
| **2** | 302 | 394 | 219 | 263 | 309 |
| **3** | 405 | 353 | 248 | 97 | 705 |
| **4** | 544 | 352 | 385 | 84 | 427 |
| **5** | 487 | 417 | 296 | 247 | 533 |
| **6** | 746 | 479 | 517 | 255 | 746 |
| **7** | 494 | 504 | 530 | 399 | 407 |
| **8** | 695 | 536 | 234 | 313 | 537 |
| **9** | 690 | 624 | 473 | 137 | 764 |
| **10 (Most deprived)** | 733 | 762 | 578 | 146 | 731 |

**Webfigure 23: European Age Standardised Mortality Rates (EASRs) per 100,000 population per year for females within religious group by deprivation (1991-2009)**

| **Deprivation decile** | **Roman Catholic** | **Church of Scotland** | **Other Christian** | **Other religion** | **No religion** |
| --- | --- | --- | --- | --- | --- |
| **1 (Least deprived)** | 127 | 241 | 223 | 140 | 253 |
| **2** | 211 | 215 | 160 | 57 | 301 |
| **3** | 290 | 236 | 154 | 71 | 236 |
| **4** | 347 | 282 | 199 | 512 | 366 |
| **5** | 370 | 281 | 218 | * | 471 |
| **6** | 358 | 327 | 280 | 516 | 256 |
| **7** | 345 | 332 | 227 | * | 358 |
| **8** | 374 | 342 | 405 | 264 | 573 |
| **9** | 403 | 406 | 446 | 769 | 708 |
| **10 (Most deprived)** | 502 | 517 | 316 | 227 | 580 |

*There were too few deaths in this group to calculate age-standardised mortality rates

**Webfigure 24 - Heat map showing RIIs in HRs (95% CI) using deprivation ranks from within population subgroups for those aged 0-64 years in April 1991 (1991-2009)**


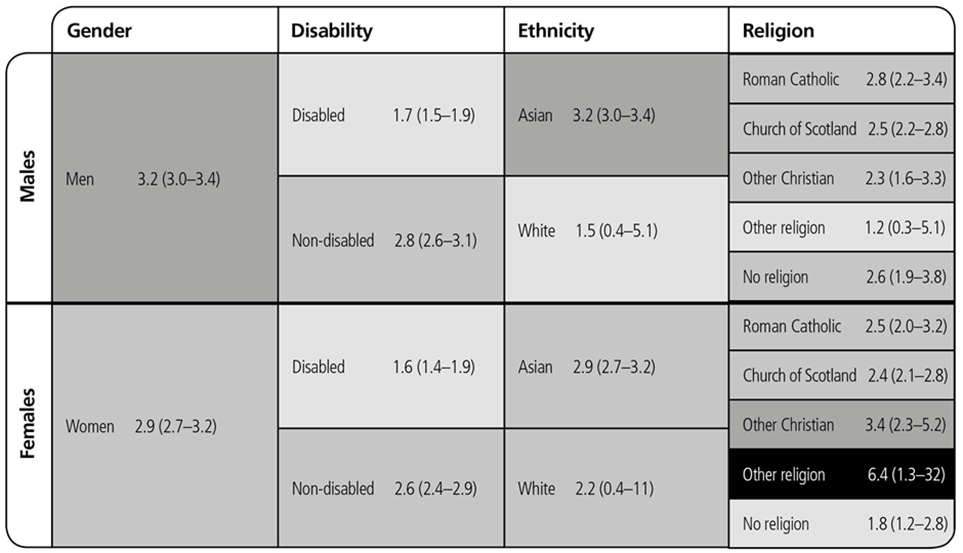


**Webfigure 25 - Heat map of RIIs of HRs (95% CIs) using social class from within population subgroups for those aged 16-64 years in April 1991 (1991-2009)**


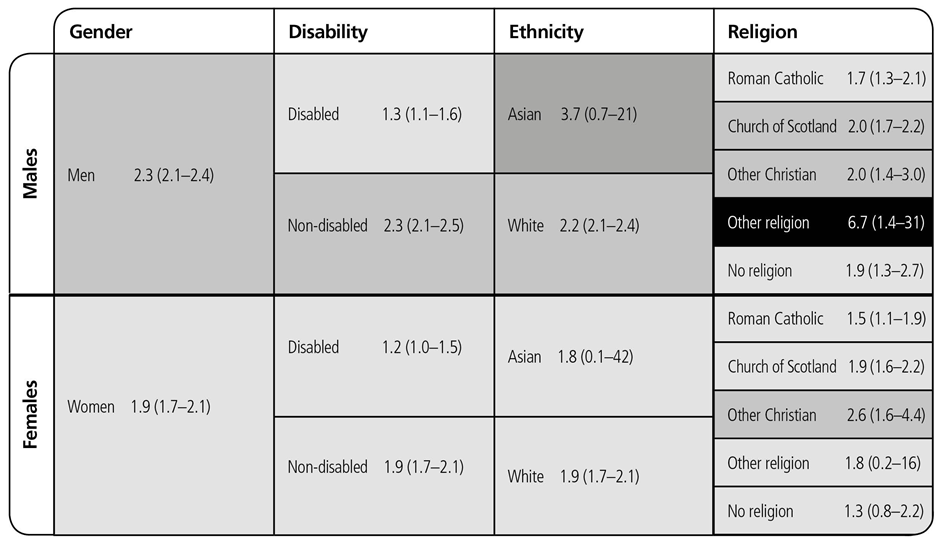

Supplement: Additional file 2: — Figures S1-S25 provide further comparisons of HRs, RIIs and EASRs. (DOCX 879 kb) [file 12939_2015_274_MOESM2_ESM.docx]
